# Energy status of ripening and postharvest senescent fruit of litchi (*Litchi chinensis* Sonn.)

**DOI:** 10.1186/1471-2229-13-55

**Published:** 2013-04-02

**Authors:** Hui Wang, Zhengjiang Qian, Sanmei Ma, Yuchuan Zhou, John W Patrick, Xuewu Duan, Yueming Jiang, Hongxia Qu

**Affiliations:** 1 Key Laboratory of Plant Resources Conservation and Sustainable Utilization, South China Botanical Garden, Chinese Academy of Sciences, Guangzhou, 510650, P R China; 2 University of Chinese Academy of Sciences, Beijing, 100049, P R China; 3 Department of Biotechnology, Jinan University, Guangzhou, 510632, P R China; 4 Australian Institute for Bioengineering and Nanotechnology, the University of Queensland, Brisbane St Lucia, QLD, 4072, Australia; 5 School of Environmental & Life Sciences, the University of Newcastle, Callaghan, NSW, 2308, Australia

## Abstract

**Background:**

Recent studies have demonstrated that cellular energy is a key factor switching on ripening and senescence of fruit. However, the factors that influence fruit energy status remain largely unknown.

**Results:**

HPLC profiling showed that ATP abundance increased significantly in developing preharvest litchi fruit and was strongly correlated with fruit fresh weight. In contrast, ATP levels declined significantly during postharvest fruit senescence and were correlated with the decrease in the proportion of edible fruit. The five gene transcripts isolated from the litchi fruit pericarp were highly expressed in vegetative tissues and peaked at 70 days after flowering (DAF) consistent with fruit ADP concentrations, except for uncoupling mitochondrial protein 1 (*UCP1*), which was predominantly expressed in the root, and ATP synthase beta subunit (*AtpB*), which was up-regulated significantly before harvest and peaked 2 days after storage. These results indicated that the color-breaker stage at 70 DAF and 2 days after storage may be key turning points in fruit energy metabolism. Transcript abundance of alternative oxidase 1 (*AOX1*) increased after 2 days of storage to significantly higher levels than those of *LcAtpB*, and was down-regulated significantly by exogenous ATP. ATP supplementation had no significant effect on transcript abundance of ADP/ATP carrier 1 (*AAC1*) and slowed the changes in sucrose non-fermenting-1-related kinase 2 (*SnRK2*) expression, but maintained ATP and energy charge levels, which were correlated with delayed senescence.

**Conclusions:**

Our results suggest that senescence of litchi fruit is closely related with energy. A surge of *LcAtpB* expression marked the beginning of fruit senescence. The findings may provide a new strategy to extend fruit shelf life by regulating its energy level.

## Background

Fruit ripening and senescence are active processes initiated by internal and environmental factors. Considerable evidence suggests that cellular energy supply is a key factor controlling ripening and senescence events, and that aging and browning of postharvest horticultural crops may be related to inadequate supplies and reduced efficiency of cellular energy generation [1-3]. A salient feature initiating the aging process is a decline in adenosine triphosphate (ATP) levels. For instance, increased membrane permeability and enhanced reactive oxygen species (ROS) production by harvested fruit are related to low ATP and energy charge (EC) levels [4,5]. Preharvest application of boron (B) and calcium (Ca) [6], and postharvest treatments, including ethylene inhibitors [7], high oxygen [4], anaerobic conditions [8], controlled atmosphere (CA) storage [2,3,5,9], an exogenous carbon source [1] and ATP [10,11], can maintain tissue levels of ATP and EC, thereby delaying pericarp browning of litchi and longan and internal flesh browning in 'Conference' pears. In addition, exogenous ATP elevates ATP levels, inhibits ROS accumulation, and maintains unsaturated fatty acid levels and membrane integrity, thus delaying senescence and deterioration of horticultural products [5,7-10,12-14]. Therefore, browning and senescence are closely related with cellular energy status, and measures to maintain energy levels can delay aging and inhibit occurrence of browning and deterioration to a certain extent. However, the mechanism contributing to the energy deficit remains unclear owing to the diversity of energy regulatory elements and complexity of energy production, transfer and control. Moreover, a growing body of evidence indicates that cellular energy levels are closely related to the activity, transcription and translation of certain regulatory elements [15-18]. Among these regulatory elements, proteins that synthesize (ATP synthase), dissipate (alternative oxidase, AOX; and mitochondrial inner membrane uncoupling proteins, UCP), transport [adenosine diphosphate (ADP)/ATP carrier, AAC] and regulate (sucrose non-fermenting-1-related kinase, SnRK) ATP have attracted considerable attention.

F1F0-ATP synthase is a multimeric enzyme that catalyzes the final step of oxidative phosphorylation and photophosphorylation, the synthesis of ATP from ADP and inorganic phosphate (P_i_) [19,20]. The general structure of the core subunits of the enzyme are highly conserved in both prokaryotic and eukaryotic organisms, and are designated as αβγδε [16]. As a novel cell death regulator, subunit β (AtpB) plays a pivotal role in stress responses. Up-regulation of *AtpB* transcription marks initiation of the aging process [21,22]. Complex V cannot be assembled successfully and respiration rates are reduced resulting in inhibition of ATP synthesis in the absence of the β subunit [21]. However, it is unknown whether *AtpB* acts as a senescence marker gene in fruit as in photosynthetic tissues.

AOX and UCP are ubiquitous in plants and have various physiological roles. These include heat production and protection against oxygen free radicals mediating the non-phosphorylating bypasses that affect ATP synthesis [16,23-26]. In plants UCP and AOX belong to multigene families, of which members exhibit specific organ and temporal expression patterns that respond differentially to stress conditions [27]. AOX expression and activity are generally induced under stress conditions, and consequently AOX is used as a marker for cell reprogramming under stress [28-31]. The alternate oxidation pathway may slow down the aging process and quality deterioration of postharvest horticultural crops [29]. Furthermore, changes in AOX and UCP accumulation in vine-ripened fruit differ significantly from those of postharvest-ripened fruit [32,33]. This implies that a complementary relationship exists between the two energy dissipation pathways. Several reports have described the complex regulation of AOX and UCP at the translational and posttranslational levels. However, most research on these two genes has focused on climacteric fruit, such as tomato, apple, banana, and mango [34-37], and their roles in non-climacteric fruit remains unclear.

ADP/ATP carrier (AAC) belongs to the high-abundance mitochondrial transporter protein family [38]. It catalyzes a counter-exchange of cytosolic ADP with matrix ATP, enabling mitochondria to supply the cytosol, and subsequently other organelles, with energy [39]. In higher organisms AAC is the core protein of the mitochondrial adenine nucleotide translocator and the main regulator of mitochondrial ATP concentration [15,40,41]. As ‘the energy regulator’ in the cell, sucrose non-fermenting-1-related protein kinase (SnRK) can sense the internal energy status by measuring cytosolic ATP/ [adenosine monophosphate (AMP) ] ratios and controlling the expression and phosphorylation of key metabolic enzymes [15]. However, to the best of our knowledge, no report has documented these two genes in the development and senescence of fruit.

Overall, in contrast to the relatively well-known physiological roles of these genes in stress responses, little information is available on how these genes contribute to the energy status of developing and senescing fruit. Studying the transcript level of these genes in relation to cellular energy status is crucial to identify causal agents contributing to energy deficits and their role in fruit ripening and senescence. This knowledge may provide opportunities to develop new strategies to control fruit ripening and the aging process and ultimately extend the storage life of harvested fruit.

Litchi is a non-climacteric, tropical and subtropical fruit with a high commercial value on the international market. Litchi fruit deteriorate rapidly after harvest because of water loss, pericarp browning and rot development [42]. Our previous studies reported that an exogenous ATP supply enhanced antioxidant systems and maintained membrane integrity to delay browning and senescence of litchi fruit [11,13,14]. However, the molecular mechanisms underlying these phenomena remain unclear. In the current study, full-length sequences of *AtpB*, *AOX1*, *UCP1*, *AAC1* and *SnRK2* were cloned from litchi fruit. Transcript abundance of these energy-related genes, respiration intensity and fruit energy status were determined for developing and postharvest-senescent litchi fruit.

## Results

### Preharvest

#### Growth curve and pericarp color of litchi fruit

**Figure 1:**
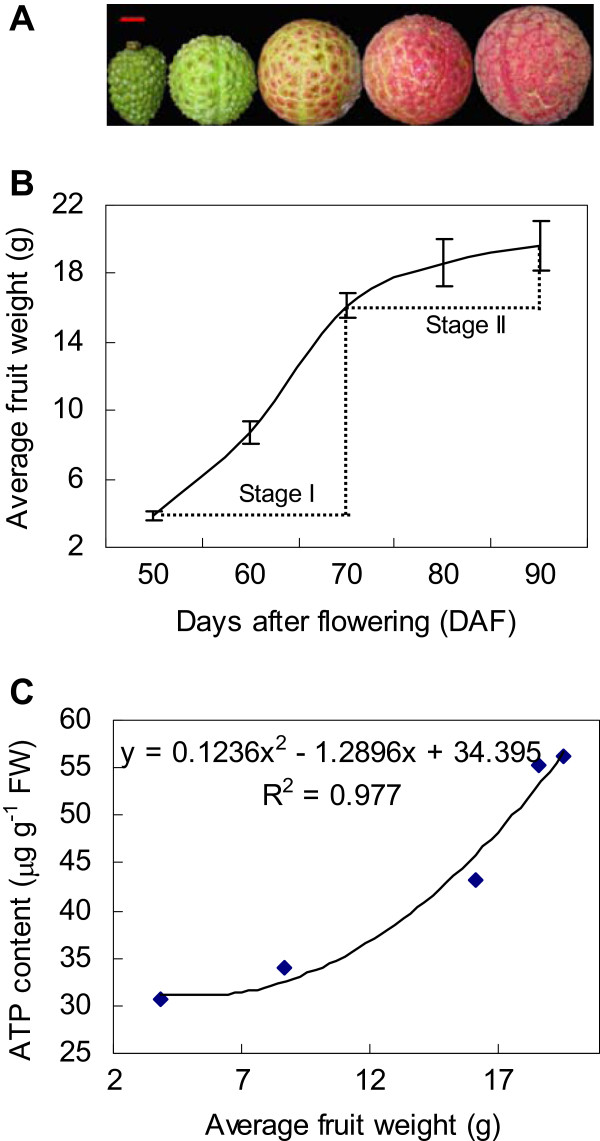
**Appearance (A), average fruit weight (B), and the correlation between average fruit weight and ATP level (C) during development and ripening of litchi fruit.** Left to right: fruit at 50 DAF, 60 DAF, color-breaker stage at 70 DAF, red-ripe stage at 80 DAF, and over-ripe stage at 90 DAF. Bar = 1 cm; DAF, days after flowering; FW, fresh weight. Data are the mean ± standard deviation (*n* = 20 in B; *n* = 3–5 in C).

Figure 1A shows the appearance of fruit at five developmental stages ranging from 50 days after flowering (DAF) (young) to 90 DAF (fully ripe) at 10-day intervals. Fruit growth was divided into two stages. Stage I (50-70 DAF) represented a rapid growth phase, whereas stage II (70-90 DAF) was a slow growth phase as fruit approached maturity (Figure 1B). Lightness values significantly increased during stage I (50-70 DAF), but chroma values increased more markedly at 50-60 DAF, which indicated that color intensity rose before it decreased (Table 1). Hue angle decreased slowly at 50-60 DAF, but more substantially from 60 to 80 DAF before attaining a plateau (Table 1), which was consistent with the change in color of the pericarp (Figure 1A).

**Table 1:** Color parameters (lightness, chroma, and hue angle) of litchi fruit at different development and ripening stages

**Color parameters**	**Days after flowering (DAF)**				
	**50**	**60**	**70**	**80**	**90**
Lightness	45.60 ± 1.36	47.93 ± 1.39	51.99 ± 2.77	43.14 ± 2.23	36.48 ± 2.72
Chroma	32.87 ± 1.09	38.34 ± 0.95	36.03 ± 1.15	34.63 ± 0.94	28.21 ± 1.52
Hue angle	115.19 ± 0.79	109.74 ± 1.37	75.94 ± 6.60	36.67 ± 2.91	34.62 ± 5.50

Data are the mean ± standard deviation (n = 15–20).

#### Respiration intensity, oxygen consumption rate and cyanide-resistant respiration

**Figure 2:**
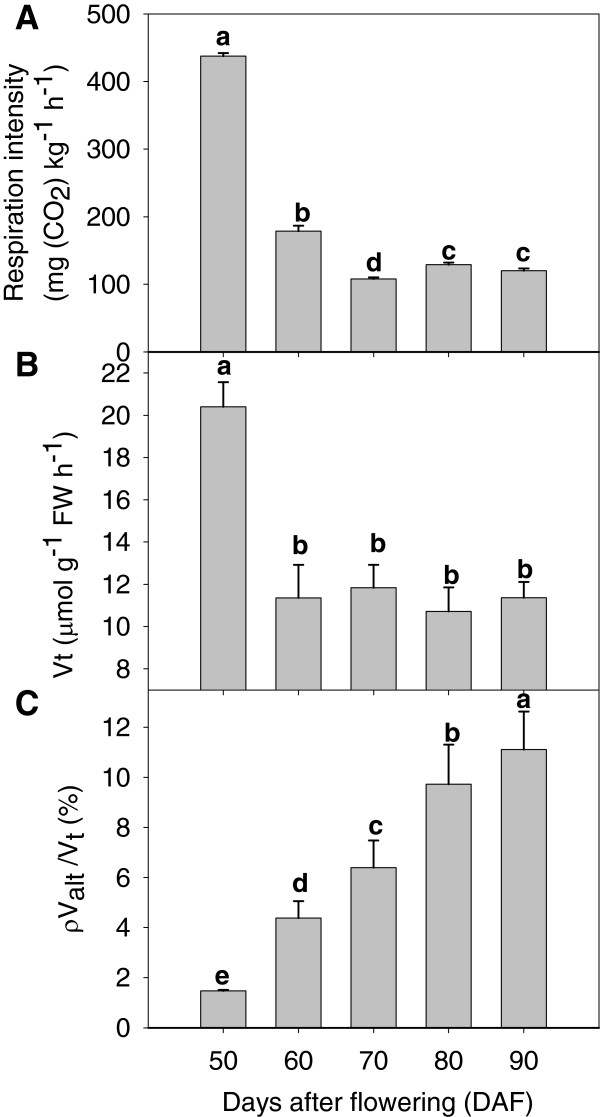
**Respiration intensity (A), *V***_***t***_**(B) and ρ *V***_***alt***_**/*V ***_***t***_**(C) during development and ripening of litchi fruit.***V*_t_, oxygen consumption rate; ρ*V*_*alt*_/*V*_*t*_, contributions of alternative oxidative respiration to total respiration. Data are the mean ± standard deviation (*n* = 3–6). Means with the same letter are not significantly different (*p* < 0.05) among the different developmental stages.

**Figure 3:**
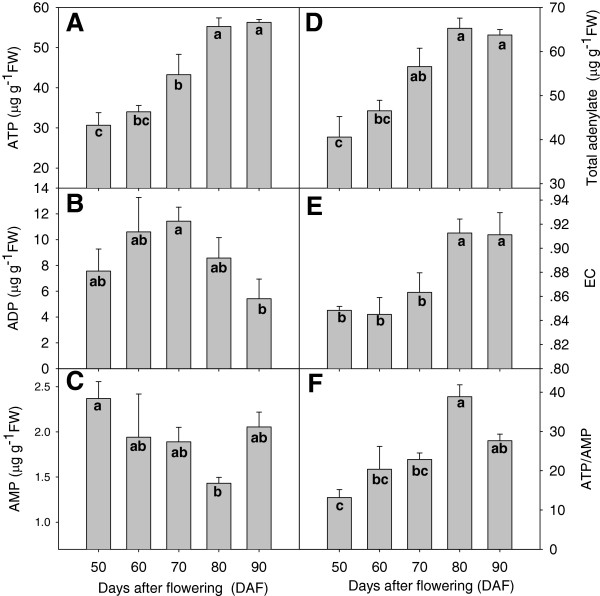
**Energy status during development and ripening of litchi fruit.** (**A**) Adenosine triphosphate (ATP); (**B**) adenosine diphosphate (ADP); (**C**) adenosine monophosphate (AMP); (**D**) total adenylate; (**E**) energy charge (EC); and (**F**) ratio of ATP/AMP. Contents of ATP, ADP, AMP and total adenylate are expressed as μg g^-1^ FW. FW, fresh weight. Data are the mean ± standard deviation (*n* = 3). Means with same letter are not significantly different (*p* < 0.05) among the different developmental stages.

Whole-fruit respiration intensity decreased significantly during stage I, but increased slightly during stage II (Figure 2A). The decrease in respiration intensity during stage I may be feedback to enhanced ADP accumulation (Figure 3B). Oxygen consumption rate (*V*_t_) declined significantly after 60 DAF and thereafter did not change (Figure 2B). Cyanide-resistant respiration capacity (the proportion of alternative oxidative respiration to total respiration, ρ*V*_alt_/*V*_t_) increased significantly during fruit development and ripening at 50–90 DAF (Figure 2C), which implies it has a pivotal role in the balance between respiration and production of ROS at these stages.

#### Energy status

ATP concentrations were correlated strongly with fruit fresh weight (Figure 1C), and increased rapidly between 50 and 80 DAF before slowing at the final stage of maturation (80–90 DAF) (Figure 3A). ADP concentrations increased during stage I but decreased progressively during stage II (Figure 3B). AMP concentrations decreased gradually throughout fruit development, but increased slightly in the last 10 days before harvest (Figure 3C). Total adenylate levels increased steadily between 50 and 80 DAF, and did not change thereafter (Figure 3D). The EC level of the fruit remained relatively constant in young fruit, and increased markedly as the fruit ripened between 80 and 90 DAF (Figure 3E). The ATP/AMP ratio peaked at 80 DAF, and thereafter declined until maturity (Figure 3F).

#### Isolation and sequence analysis of energy-related genes

Partial sequence fragments of energy-related genes were isolated by reverse transcription- PCR (RT-PCR) using degenerate primers (Table 2). Sequencing of several of these fragments revealed the existence of one isoform for each gene. These genes were *LcAtpB* (1188 bp), *LcAOX1* (676 bp), *LcUCP1* (652 bp), *LcAAC1* (850 bp) and *LcSnRK2* (773 bp). Full-length sequences of these genes were obtained after 3′ and 5′-RACE. These sequences were compared with known sequences from other species using the NCBI BLAST server. The GenBank accession codes of the sequences are listed in Table 3.

**Table 2:** Degenerate primers for cloning of energy-related genes in litchi pericarp

**Gene**	**Forward primer (5’ to 3’)**	**Reverse primer (5’ to 3’)**	**Product (bp)**
*AtpB*	GGGCCGTGGCCatgwsngcnac	TCGGGCAGGCCGtcnarytcncc	1188
*AOX1*	GGAGGACGGCACCGAntggmmntg	GCGTCCTTGGGCAGCCkccartartcda	676
*UCP1*	ACCATCGCCCGGGARGARGGNNT	AGGTGCCCAGCCGGSYRAARTTNGG	652
*AAC1*	GCCGCCGCCccnathgarmg	CGCCGCCGGAGCcrtayttyttnc	850
*SnRK2*	GGCGCCGGCAACttyggngtngc	AGCTCCCTGGGCAGGttyttnarraac	773

*AtpB*, ATP synthase β subunit; *AOX1*, alternative oxidase 1; *UCP1*, uncoupling mitochondrial protein 1; *AAC1*, ADP/ATP carrier 1; *SnRK2*, sucrose non-fermenting-1-related kinase 2.

**Table 3:** Homologies based on nucleotide sequences for energy-related genes isolated from litchi cv. Huaizhi

**Gene**	**GenBank number**	**Top *Arabidopsis *BLAST match**	**Top BLAST match excluding *Arabidopsis***	**Homology (%)**
*LcAtpB*	JQ349005	AJ271468.1 *AtAtpB*	AJ235513.2 *AtpB Koelreuteria paniculata*	60^a^, 92^b^
*LcAOX1*	JQ349006	NM_113135.3 *AtAOX1A*	EF523518.1 *AOX1a Nicotiana glutinosa*	69^a^, 71^b^
*LcUCP1*	JQ349009	NM_125287.4 *AtUCP2*	XM_002520396.1 *UCP Ricinus communis*	66^a^, 65^b^
*LcAAC1*	JQ349007	NM_121352.3 *AtAAC2*	XM_002531865.1 *AAC Ricinus communis*	71^a^, 75^b^
*LcSnRK2*	JQ349008	NM_001203118.1 *AtSnRK2.2*	XM_002513909.1 *SAPK1 Ricinus communis*	68^a^, 73^b^

See legend for Table 2 for names of genes.

The coding region of *LcAtpB* was 1377 bp in length, encoding a deduced 459-amino acid (aa) sequence, with a predicted MW of 49.90 kDa and a calculated isoelectric point (pI) of 5.12. LcAtpB was 92% homologous with the AtpB from *Koelreuteria paniculata,* a close relative of litchi*.* The C-terminal domain of the β subunit contained the highly conserved ‘DELSEED’ motif (Additional files 1 and 2) involved in mechanochemical coupling of ATP synthase [43]. *AOX* belongs to a multigene family in many plants, including mango, tomato and other horticultural crops. Two types of *AOX* genes are present in angiosperms [44], of which isoforms may be differentially expressed in fruit and other tissues, such as in tomato [45]. While in the present study, transcript evidence showed support for a single predicted isoform (*LcAOX1)* for all candidates in litchi fruit pericarp and consequently only this isoform was detected during experimental validation. *LcAOX1* (1032 bp), encoding a 344 aa sequence (predicted MW 39.13 kDa; pI 8.76), showed 71% identity with that of *Nicotiana glutinosa*. Site-directed mutagenesis has shown that several residues are required for AOX activity [44]. The highly conserved residues were identified in litchi fruit using multiple sequence alignments of AOX proteins from diverse organisms (Additional files 3 and 4). UCP family from diverse organisms are grouped into five subfamilies, plant UCP1 and UCP2 belong to subfamilies II. Moreover, investigations have identified 1 to 6 genes that encode UCPs in different plants [27]. The major feature of UCPs from plants is the presence of three energy transfer protein signatures (ETPS) [23] conserved in all UCP isoforms (Additional file 5). In the present study, three copies of the ETPS signature were identified in *LcUCP1*, which contained an open reading frame (ORF) of 915 bp encoding a 305 aa protein, with a predicted MW of 33.23 kDa and a pI of 9.01. A BLAST search of GenBank revealed that LcUCP1 shared 66% identity with AtUCP2 (NM_125287.4) from *Arabidopsis thaliana* and 65% identity with RcUCP (XM_002520396.1) from *Ricinus communis* at the protein level. A neighbor-joining cladogram further showed that *LcUCP1* was most similar to *UCP* from *R. communis* (Additional files 5 and 6). There are four groups of mitochondrial adenine nucleotide carriers presented in the five eukaryotic clades, among which the ADP/ATP carriers are the best known. Furthermore, three mitochondrial AAC genes: *AtAAC1*, *AtAAC2*, and *AtAAC3* were presented in the genome of *A. thaliana*[46]. In the present study, *LcAAC1* gene isolated from litchi fruit pericarp contained an ORF of 1266 bp encoding a 422 aa protein (predicted MW 46.00 kDa; pI 9.79), and exhibited 71% identity with AAC2 from *A. thaliana* and 75% identity to AAC from *R. communis*. LcAAC1 contained a highly conserved RRRMMM signature motif (Additional files 7 and 8) [17]. On the basis of sequence similarity, domain structure and cellular function, the plant SnRK family can be divided into three subfamilies: SnRK1, SnRK2 and SnRK3. Among these subfamilies, SnRK2 is unique to plants and is involved in responses to environmental stresses [47]. In the present study, *SnRK* cDNA isolated from litchi pericarp was 1394 bp in length with a 1014 bp ORF encoding a predicted polypeptide of 338 aa (MW 38.00 kDa; pI 5.36). The deduced amino acid sequence showed homology with counterpart SnRK2 family members, namely *R. communis*, *Glycine max*, *Zea mays*, and *A. thaliana*. Similar to other *SnRK2* genes, the N-terminal catalytic domain was highly conserved, containing an ATP-binding site (DIGSGNFGVAKLVRDTWTDELLAVK) and protein kinase activating signature (QICHRDLKLENTLL), whereas the C-terminal regulatory domain contained an acidic ‘patch’, which is essential for kinase activity (Additional file 9). In a neighbor-joining cladogram *LcSnRK2* and its counterparts *OsSAPK3*, *ZmSnRK2.2*, *GmSARK2*, and *RcSAPK1* were clustered in subclass 2 (Additional file 10) as reported by Zhang et al. [47].

#### Spatial and temporal expression of energy-related gene transcripts

**Figure 4:**
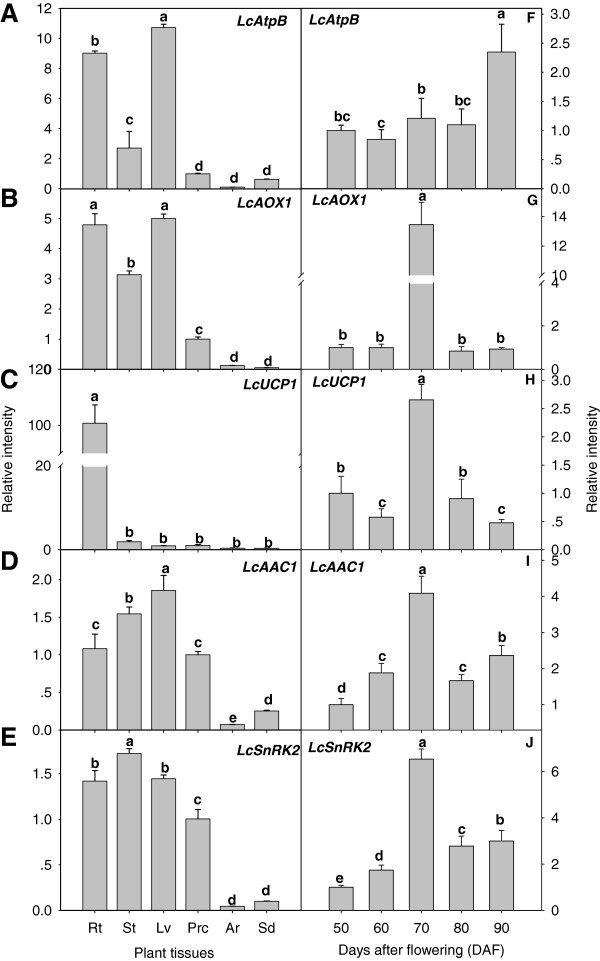
**Spatial and temporal expression of gene transcripts.** Total RNA was isolated from litchi plants 80 days after flowering for tissue-specific expression analysis. Transcript abundance was determined using qRT-PCR and was normalized using *LcACTIN*. Values are the mean ± standard deviation (*n* = 3) from three separate RNA extractions. Means with the same letter are not significantly different (*p* < 0.05) among the different tissues (**A**, **B**, **C**, **D**, and **E**) and among developmental stages (**F**, **G**, **H**, **I**, and **J**). *AtpB*, ATP synthase β subunit; *AOX1*, alternative oxidase 1; *UCP1*, uncoupling mitochondrial protein 1; *AAC1*, ADP/ATP carrier 1; *SnRK2*, sucrose non-fermenting-1-related kinase 2. Rt, root; St, stem; Lv, leaf; Prc, pericarp; Ar, aril; Sd, seed.

Expression analysis of energy-related genes indicated ubiquitous expression in all tissues tested (Figure 4). The most notable features of the tissue expression profiles were that *LcUCP1* (Figure 4C) exhibited strongest expression in the root in which transcript levels were some 100-fold greater than those detected in the other tissues. In contrast, the remaining genes exhibited high expression in vegetative tissues (root, stem, and leaf). *LcAtpB* and *LcAAC1* were expressed most strongly in the leaf (Figure 4A and D). *LcAtpB* and *LcAOX1* showed the highest expression in the root and leaf, with lower levels of expression detected in the stem, and weakest expression was observed in the fruit pericarp, aril and seed (Figure 4A and B). Thus, *LcSnRK2* exhibited strongest expression in the stem, root, leaf, and pericarp, and weakest expression in the aril and seed (Figure 4D and E).

Expression of *LcAtpB* was up-regulated by 114% at 80–90 DAF (Figure 4F), which was consistent with changes in ATP concentration (Figure 3A). Increased transcript abundance of *LcAOX1* peaked at 14-fold at 70 DAF but significant differences in transcript abundance at other stages of fruit development were not observed (Figure 4G). Abundance of *LcUCP1* declined by 43% between 50 and 60 DAF before increasing by 363% at 70 DAF and thereafter declining rapidly by 48% at 90 DAF (Figure 4H). *LcAAC1* and *LcSnRK2* exhibited similar changes in transcript levels (Figure 4I and J). Transcription of these two genes increased significantly between 50 and 70 DAF before decreasing to the lowest levels between 70 and 80 DAF, but recovered partly in the final stage of maturation (80–90 DAF).

### Postharvest

#### Disease incidence, pericarp browning, proportion of edible fruit, and membrane permeability

**Figure 5:**
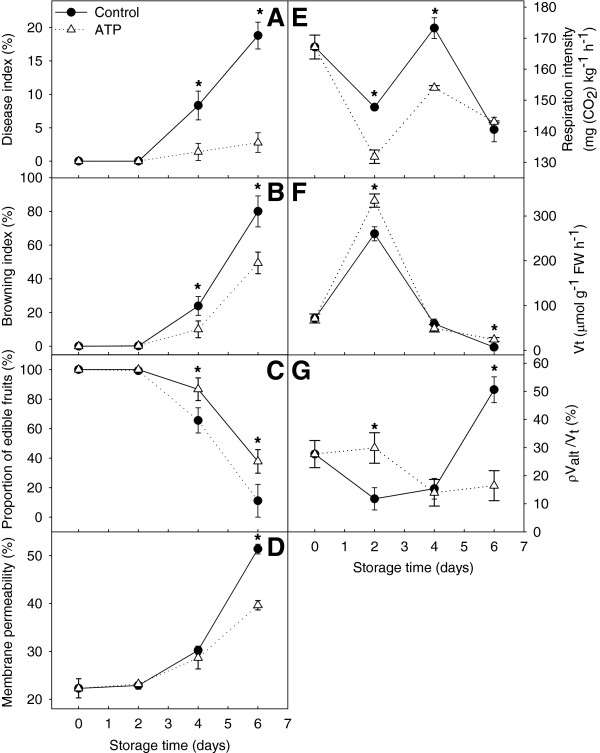
**Senescence parameters and respiration activities of harvested litchi fruit.** (**A**) Disease index; (**B**) browning index; (**C**) proportion of edible fruits; (**D**) membrane permeability; (**E**) respiration intensity; (**F**) *V*_*t*_; and (**G**) ρ*V*_*alt*_/*V*_*t*_. Data are the mean ± standard deviation (*n* = 3). An asterisk represents a significant difference (*p* < 0.05) between ATP-treated fruit and the untreated control.

The fruit disease and pericarp browning indices increased after harvest, whereas the proportion of edible fruit decreased. Exogenous ATP treatment reduced disease incidence and delayed tissue browning of the fruit significantly (Figure 5A and B). The disease and browning indices were 19% and 80% in the non-ATP-treated (control) fruit, but only 3% and 49% in ATP-treated fruit, by the end of storage. The proportion of edible fruit significantly increased after ATP treatment, by 21% and 28% compared with control fruit, after 4 and 6 days of storage, respectively (Figure 5C). Membrane permeability reflects senescence and deterioration of plant tissue and is usually measured as relative conductivity. This parameter increased in harvested fruit with no significant difference between ATP-treated and control fruit observed during 0–4 days of storage, but was significantly lower in ATP-treated fruit at the end of storage. These results suggested that exogenous ATP treatment protected the membrane system and, thus, delayed senescence and deterioration of litchi fruit in storage (Figure 5D).

#### Respiration intensity, oxygen consumption rate, and cyanide-resistant respiration

Respiration intensity fluctuated and was significantly blocked by ATP treatment (Figure 5E). The oxygen consumption rate peaked after 2 days of storage and was enhanced significantly by ATP application (Figure 5F). In the first 2 days of storage ρ*V*_alt_/*V*_t_ decreased significantly, then rose slowly during the medial period of storage and increased sharply by the end of storage; exogenous ATP treatment significantly slowed down these effects (Figure 5G). In the ATP-treated fruit ρ*Valt*/*Vt* was relatively stable throughout the storage process, which indicated that stability in ρ*Valt*/*Vt* played an important role in maintenance of fruit storability.

#### Energy status

**Figure 6:**
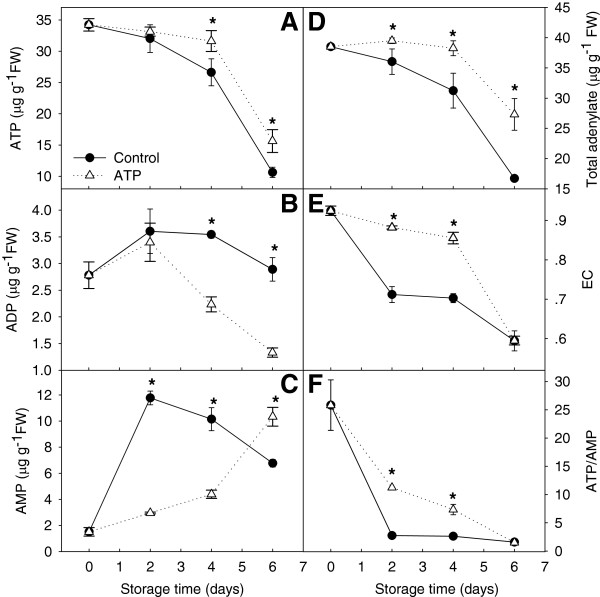
**Energy status in harvested litchi fruit.** (**A**) Adenosine triphosphate (ATP); (**B**) adenosine diphosphate (ADP); (**C**) adenosine monophosphate (AMP); (**D**) total adenylate; (**E**) energy charge (EC); and (**F**) ratio of ATP/AMP. Contents of ATP, ADP, AMP and total adenylate are expressed as μg g^-1^ FW. FW, fresh weight. Data are the mean ± standard deviation (*n* = 3). An asterisk represents a significant difference (*p* < 0.05) between ATP-treated fruit and untreated control.

The ATP concentration significantly decreased by 69% during 6 days of storage, and the severity of the decline was reduced in ATP-treated fruit (Figure 6A). No significant difference in ADP concentration was observed during the 6 days of storage, whereas ATP treatment reduced ADP levels by 37% compared to that of control fruit after 4 days of storage (Figure 6B). The AMP concentration significantly increased during the first 2 days of storage before decreasing as storage progressed, and ATP treatment significantly depressed (by 75%) AMP levels (Figure 6C). The total adenylate concentration decreased significantly during postharvest storage and was 57% lower by the end of storage, but was up-regulated by ATP treatment (Figure 6D). Energy charge showed the same pattern but no significant difference in EC level between control and ATP-treated fruit was observed by the end of storage (Figure 6E). The ATP/AMP ratio decreased significantly after harvest. ATP treatment significantly attenuated the decrease in the ATP/AMP ratio until 6 days of storage (Figure 6F).

#### Abundance of energy-related gene transcripts

**Figure 7:**
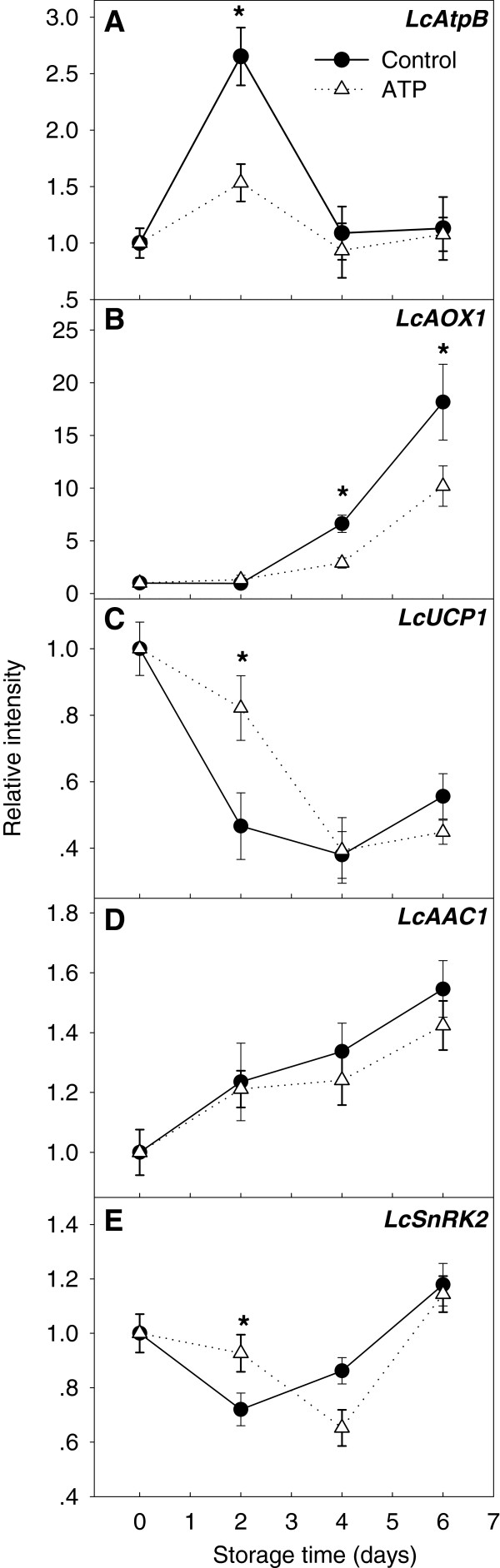
**Relative intensity of gene transcripts in harvested litchi fruit.** Transcript abundance was determined using qRT-PCR and was normalized using *LcACTIN*. Data are the mean ± standard deviation (*n* = 3). An asterisk represents a significant difference (*p* < 0.05) between ATP-treated fruit and untreated control. See Figure 4 legend for names of genes.

*LcAtpB* expression significantly increased and peaked 2 days after harvest before declining, whereas exogenous ATP treatment blocked the expression peak at 2 days postharvest (Figure 7A). *LcAOX1* transcript abundance remained stable until 2 days of storage, and thereafter increased constantly to 17-fold higher than the initial level at 6 days of storage. Exogenous ATP significantly inhibited the rise in *LcAOX1* transcript level after 4 days of storage (Figure 7B). The *LcUCP1* transcript level declined during the first 4 days of storage, with a slight increase observed thereafter, and ATP slowed both of these changes (Figure 7C). The *LcAAC1* transcript level rose slowly after harvest and no marked effect was imposed by exogenous ATP application (Figure 7D). The transcript abundance of *LcSnRK2* decreased within the first 2 days of storage and subsequently increased, and ATP slowed both of these changes (Figure 7E).

## Discussion

### Preharvest

#### ATP level increased and energy charge remained relatively constant during fruit development and ripening

Changes in energy levels may be a key factor in switching on fruit ripening and senescence. Initiation of senescence is characterized by a reduced ATP level [48]. In the present study, ATP, EC, and total adenylate levels and the ATP/AMP ratio increased rapidly, whereas the AMP concentration decreased gradually, during fruit development (50–80 DAF) (Figure 3). ATP is produced mainly by oxidation of carbohydrates and is regulated by transcription of key proteins catalyzing nucleotide synthesis [15]. The respiratory rate of pericarp tissue of litchi fruit declined during fruit development, whilst the cyanide-resistant respiration to total respiration ratio (ρ*V*_alt_/*V*_t_) and energy production were enhanced (Figures 2 and 3). A declining respiratory rate and enhanced alternative respiration is an overflow protection mechanism to prevent ROS production [49].

#### Color-breaker stage at 70 DAF may be a key turning point in fruit energy metabolism

We isolated total RNAs from the root, stem, leaf, pericarp, aril and seed from fruit 80 DAF for real-time RT-PCR analysis. Five gene transcripts were predominantly expressed in the root, stem, and leaf at this specific developmental stage and increased significantly in developmental stage I except for *LcUCP1*, which was predominantly expressed only in the root, and *LcAtpB*, which was up-regulated by the end of developmental stage II. The *LcAtpB* expression level rose continuously during fruit development and maturation consistent with the increased ATP concentration (Figure 3A). Transcript abundance of *LcAOX1*, *LcUCP1*, *LcAAC1*, and *LcSnRK2* reached peaks at 70 DAF in the rank order *LcAOX1* >*LcSnRK2* >*LcAAC1* >*LcUCP1* (Figure 4F–J). Their transcription peaks coincided with the peak in ADP concentration (Figure 3D). Transcription peaks of *AOX1b* and *UCP* were also observed in mango fruit at the turning stage [35], which indicated that the color-breaker stage may be a key turning point in fruit energy metabolism. *LcAOX1*, *LcUCP1*, *LcAAC1* and *LcSnRK2* transcript levels significantly decreased after 70 DAF, allowing a surge in ATP synthesis. Under the combined effects of *AtpB*, *AOX1*, *UCP1*, *AAC1*, and *SnRK2*, the EC level remained relatively constant during fruit development and ripening. *LcAOX1* expression spiked at 70 DAF, which probably reduced levels of ROS generated by high rates of fruit photosynthesis. Recent studies indicate that the SnRK2 family plays key roles in stress responses, such as hyperosmotic stress and abscisic acid (ABA) signaling. ABA is involved in the maturation of many climacteric and non-climacteric fruit. Commonly, endogenous ABA concentrations increase during fruit maturation, whereas exogenous application of ABA accelerates fruit ripening or maturation [50]. Sugar signaling also regulates senescence in a complex network with other signals [47] resulting from biotic or abiotic stress. SnRK2 has been implicated in stress and ABA-mediated signaling pathways [51]. Enhanced transcript abundance of *LcSnRK2* at 70 DAF might be a consequence of increased ABA and sugar accumulation during maturation of litchi fruit [50].

### Postharvest

#### ATP and energy charge levels decreased significantly in postharvest litchi fruit

The levels of ATP, total adenylate, and EC, and the ATP/AMP ratio significantly decreased, but the AMP concentration significantly increased, in stored harvested fruit (Figure 6). Similar results were reported for apples, pears, cut flowers, and other horticultural products [1-3]. ATP content decreased significantly in ‘Jonagold’ apples during controlled atmosphere storage. Brown heart disease of ‘Conference’ pears during storage was partly attributed to energy depletion [9]. As the petals of carnation cut flowers wilt (a senescence symptom), ATP synthesis decreases [1,2]. ATP concentrations were negatively correlated with the browning index and proportion of edible fruit, which was consistent with our previous work [7]. ATP and EC levels in horticultural crops can be maintained by appropriate handling, thus effectively postponing senescence. Application of ethylene inhibitors, high oxygen, anaerobic conditions, exogenous sucrose, or ATP can maintain the ATP content and EC levels in litchi, longan, tulip and carnation flowers, thereby delaying pericarp browning and extending flowering longevity [1,3,4,7,10]. In the present study, the decrease in ATP, total adenylate, and EC levels and the ATP/AMP ratio were significantly slowed by exogenous ATP. At the same time, the disease and browning indices were lower and commodity rates were higher in ATP-treated fruit (Figure 5, Additional file 11). These data support the hypothesis that intracellular energy depletion or ATP deficiency induce senescence of horticultural crops. Respiratory intensity decreased in harvested litchi fruit but increased rapidly during 2–4 days in storage. Respiration rates might be activated by ADP accumulated during the first 2 days of fruit storage (Figure 6B). Energy levels, oxygen consumption rate, and ρ*V*_alt_/*V*_t_ were increased by exogenous ATP treatment. A reduced respiration rate is apparently favorable to extend the storage life of litchi fruit. The highly flexible nature of respiratory metabolism in plants allows survival under varying and often stressful environmental and nutritional conditions [52]. In the present study, exogenous ATP treatment extended the storage life of litchi fruit by reducing respiration intensity, which differed from the results of Klotz et al. [53] that suggested adenylates did not limit respiration in stored sugarbeet roots.

#### Decrease in ATP and energy charge levels may have resulted from imbalanced accumulation of LcAOX1 and LcAtpB transcripts in postharvest litchi fruit

*LcAtpB* transcription was up-regulated in harvested litchi fruit, which might restrain the rapid decline in ATP level during 0–4 days of storage. Exogenous ATP treatment inhibited *LcAtpB* expression, possibly by feedback inhibition by ATP. Chivasa [21] reported a new role for *AtpB* as a pro-cell death protein. In the current study, in preharvest (80–90 DAF) or postharvest (0–2 days in storage) litchi fruit, the rapid increase of *LcAtpB* expression followed by rapid senescence and decline in fruit quality suggested that the increase in *LcAtpB* transcript level marked initiation of the aging process. During abiotic stresses, such as heat shock and hypoxia, AMPKs (SnRK analogues) switch off ATP-consuming processes and activate ATP-generating catabolic pathways through direct enzyme regulation and transcriptional regulation [54]. SnRK may sense ATP deficiency and trigger downstream transcription of *AtpB*[55]. In the present study, the transcript abundance of *LcAtpB* was significantly increased in postharvest litchi fruit, which might be regulated by *LcSnRK2*.

**Figure 8:**
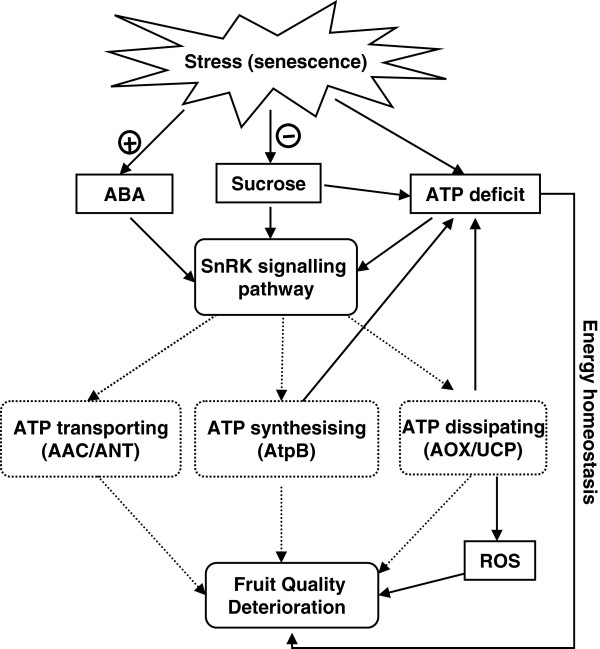
**Possible mechanism to account for energy regulation in senescent litchi fruit.** An energy deficit in intensive respiration during the senescence process may be sensed by sucrose non-fermenting-1-related kinase (SnRK), which controls the expression and phosphorylation of key metabolic enzymes, and this might involve ATP synthase, ADP/ATP carrier (AAC), alternative oxidase (AOX) and uncoupling mitochondrial protein (UCP). The energy homoeostatic condition can be maintained to a certain extent, but the equilibrium is ultimately disrupted, which is correlated with fruit deterioration.

UCP and AOX energy-dissipating systems play similar roles in mitochondrial energy-linked processes in plants, either through tissue-specific thermogenesis or by protecting plant cells against oxidative stress. The initial steps of cellular death are accompanied by an oxidative burst, depletion of ATP, and a strong stimulation of AOX expression [56]. In the present study, the transcript abundance of *LcAOX1* in litchi fruit was significantly up-regulated during storage. The lower ATP level in control fruit might have resulted from enhanced transcription and activity of AOX. Mitochondrial inner membrane UCPs catalyze a proton conductance that dissipates the proton electrochemical gradient established by the respiratory chain, thus affecting ATP synthesis. *LcUCP1* continued to decline in the first 4 days of storage, but thereafter rebounded slightly. Differential expression and complementary function of AOX and UCP is reported in a variety of fruits, vegetables, and plant tissues, and may be regulated by ethylene, low temperature, and other factors [27,31,33-35,57,58] through different catalytic efficiencies under distinct physiological conditions [26]. ADP/ATP carrier (AAC) is unable to modify the net content of adenine nucleotides (ATP + ADP + AMP) because it always exchanges one nucleotide for another, whereas mutation of the AAC signature sequence (Arg triplet) inhibits ATP synthesis in cells and isolated mitochondria. In addition, loss of endogenous nucleotides is responsible for impeding ADP/ATP transport [17,39]. In the present study, *LcAAC1* was up-regulated, and its transcript abundance negatively correlated with a decreased adenylate pool, in litchi fruit during storage. Furthermore, *LcAAC1* was not significantly affected by exogenous ATP treatment (Figures 6D and 7D), which indicated that *LcAAC1* was not sensitive to adenylate content. Transcript abundance of *LcAtpB*, *LcAOX1*, and *LcAAC1* were significantly up-regulated, whereas *LcUCP1* and *LcSnRK2* expression decreased before being enhanced, in harvested fruit and ATP supplementation slowed all these changes (Figure 7). Moreover, *LcAOX1* transcript abundance was significantly higher than that of *LcAtpB.* Under the joint action of *LcAtpB*, *LcAOX1*, *LcUCP1*, *LcAAC1*, and *LcSnRK2*, the EC remained relatively balanced between 2 and 4 days of storage. However, the dynamic energy equilibrium was eventually broken, probably because of dramatic increases in the transcript level of *LcAOX1* after 4 days of storage, causing a significant decrease in ATP and EC levels by the end of storage, which was correlated with rapid fruit deterioration (Figure 8).

## Conclusions

This study investigated energy characteristics and the level of five transcripts that are involved in the modulation of ATP levels during preharvest development and postharvest senescence of litchi fruit. Measurements were also recorded after ATP treatment of harvested fruits. Senescence and deterioration of litchi fruit during storage was closely related to ATP content and EC levels. Dramatic increase in *LcAtpB* expression either in planta or postharvest suggested that *LcAtpB* expression may mark the onset of senescence of litchi fruit. The energy status in litchi fruit seems to be controlled by the combined effects of genes responsible for energy production, dissipation, transfer and regulation, in which *LcAtpB* and *LcAOX1* play a decisive role. Given their importance for energy regulation, these genes could be used to generate markers for the breeding of new litchi cultivars that show increased ATP production or fruit with a longer postharvest storage life.

## Methods

### Plant materials and treatments

Seven 10-year-old litchi (*Litchi chinensis* Sonn*.* cv. Huaizhi) trees were chosen for these experiments from a commercial orchard in Guangzhou, China, in 2010 and 2011. Ten fruit on each tree were used to measure fruit diameter every 10 days over a period of 40 days beginning at 50 DAF (31 May, 2010; 2 June, 2011) and ending at 90 DAF (11 July, 2010; 12 July, 2011). At each stage, fruit were randomly sampled from different parts of the canopy between 9 and 11 a.m. Fresh weights of individual fruit were recorded, and respiration rate was measured using fresh fruit. The pericarp was collected, frozen in liquid nitrogen and stored at -80°C for RNA extraction. All trees used in the above studies were maintained in accordance with commercial litchi production practices.

Litchi fruit at about 80% maturity (80–82 DAF) were collected from the same orchard. In order to examine the expression pattern of energy-related gene transcripts, a variety of tissues (root, stem, leaf, pericarp, aril and seed) were collected at this maturation stage in 2012. Fruit were selected for uniformity of shape, color, and absence of blemishes or disease. The fruits were surface sterilized in 0.5% sodium hypochlorite solution for 5 s before rinsing twice in sterile distilled water, and then placed in 4.2 L glass desiccators containing sterile distilled water or 1.0 mmol L^-1^ ATP solution (200 fruit L^-1^). A partial vacuum at 75 kPa was gently applied for 3 min. The vacuum-infiltrated fruits were air-dried, then packed in 0.015-mm-thick polyethylene bags (15 pieces/bag, 12 bags/treatment), and stored at 25°C and 85–90% relative humidity. Thirty fruit from different treatments were randomly sampled every 2 days after storage. Pericarp browning and disease incidence were recorded, and respiration rate and alternative oxidative respiratory activity were tested using fresh fruit.

### Color analyses

The pericarp color was measured with a Konica Minolta CR-400 Chroma Meter (Minolta, Japan) using 15 fruits immediately after picking. The meter was calibrated using the manufacturer’s standard white plate. Color was recorded in the L*, C* and h color space, and these values were measured randomly at the site opposite to the fruit suture (Table 1). L* indicated the lightness ranging from black (L* = 0) to white (L* = 100), chroma values (C*) represented the color saturation that varied from dull to vivid, lower and higher values, respectively, and hue angle (h*) referred to a color wheel, with red at an angle of 0°, yellow at 90°, green at 180°, and blue at 270°, in accordance with the method of McGuire [59].

### HPLC analysis of ATP, ADP and AMP

Extraction of ATP, ADP and AMP were conducted with the method of Ozogul et al. [60] with a minor modification. Litchi fruit pericarp (2 g) was ground in liquid nitrogen and extracted with 6 mL of 0.6 mol L^-1^ perchloric acid. The homogenate was centrifuged at 4°C and 16,000 × *g* for 10 min. The supernatant (3 mL) was quickly neutralized to pH 6.5–6.8 using 1 mol L^-1^ potassium hydroxide. The solution was diluted to 4 mL and passed through a 0.45 μm filter. The filtrate was then stored at -30°C until analyzed. ATP, ADP and AMP measurements were performed in accordance with the method of Liu et al. [61] with a Waters 2695 HPLC (Waters, Inc., Milford, MA, USA) using a Pinnacle ll-C_18_ column (4.6 × 250 mm) and an ultraviolet (UV) detector at 254 nm. Mobile phase A consisted of 0.06 mol L^-1^ dipotassium hydrogen phosphate and 0.04 mol L^-1^ potassium dihydrogen phosphate dissolved in deionized water and adjusted to pH 7.0 with 0.1 mol L^-1^ potassium hydroxide. Mobile phase B was 100% acetonitrile. Elution was carried out by a linear gradient program with 75–100% A and 0–25% B for 7 min. The flow rate was 1.2 mL min^-1^. Sample aliquots of 5 μL were injected into the HPLC. ATP, ADP and AMP concentrations were calculated according to the external standard curve and expressed on a fresh weight (FW) basis. Energy charge was calculated as: ([ATP] + 0.5 × [ADP])/([ATP] + [ADP] + [AMP]).

### Pericarp browning and disease incidence

Pericarp browning was assessed after 0, 2, 4 and 6 days of storage using the following scale by an experienced technician [11]: 0, no browning (excellent quality); I, slight browning; II, <1/4 browning; III, 1/4 to 1/2 browning; and IV, >1/2 browning (poor quality). The browning index was calculated as: Σ (browning scale × percentage of corresponding fruit within each class).

Disease development was monitored using 30 randomly selected fruits and recorded as the ratio of mean proportion (%) of the fruit surface with fungal growth to the total number of fruit. The rating scale described above for browning was also applied for disease severity [11]. The proportion of edible fruit was calculated as the percentage of the total number of fruit exhibiting a browning scale value ≤1.

### Membrane permeability

Membrane permeability was expressed as the relative electrolyte leakage. Fifteen discs (10 mm in diameter) from 30 fruits were washed three times in deionized water, dried with filter paper and then incubated for 30 min in 20 mL of 0.3 mol L^-1^ mannitol solution at 25°C. Initial electrolyte leakage rates were determined using a conductivity meter (model DDS-11A; Shanghai Scientific Instruments, Shanghai, China). Total electrolyte leakage was then determined after boiling for 20 min and cooling rapidly to 25°C. The relative leakage was expressed as the percentage of the initial electrolyte to the total electrolyte.

### Whole-fruit respiration intensity

The respiratory activities of intact litchi fruit were measured by infrared gas analysis. A sample of 10 litchi fruit was weighed before being sealed in an easy-lock food container (2.2 L in volume) at 25°C, through which CO_2_-free air was pumped. Increases in CO_2_ concentration in the container were monitored for 5 min by passing the air stream through an infrared gas analyzer (Li-6262 CO_2_/H_2_O analyzer, LI-COR, Inc., Lincoln, NE, USA). Respiration rate was expressed in terms of carbon dioxide output per hour per kilogram of fresh weight (mg CO_2_ kg^-1^ h^-1^). The analysis was carried out using five replicate fruit samples.

### Oxygen consumption rate and the contributions of alternative oxidative respiratory to total respiration

The oxygen consumption rate of fruit pericarp was measured using a Clark-type Oxygraph oxygen electrode (Hansatech Instruments, King’s Lynn, UK) at 25°C by the method of Bingham and Farrar [62] with some modifications. Ten pericarp discs (5 mm in diameter) were dipped in distilled water before surface drying with filter paper. Potassium phosphate buffer (65 mmol L^-1^, pH 6.8) was used as the respiratory medium. The total respiration rate (*V*_t_) was determined in the absence of any respiratory inhibitors. The alternative respiratory pathway (ARP) rate (ρ*V*_alt_) was calculated from *V*_t_ minus the respiration rate in the presence of 25 mmol L^-1^ benzohydroxamate. The contribution of ARP to total respiration was expressed as ρ*V*_alt_/*V*_t_. The oxygen consumption rate was expressed as nmol min^-1^ cm^-2^.

### RNA extraction

Total RNA was extracted from litchi fruit pericarp using the hot borate method of Won and Wilkins [63], with some modifications as described by Kuang et al. [64]. Extracted RNA was digested with DNase I (TaKaRa Bio, Inc., Otsu, Shiga, Japan) and then confirmed by PCR to be free of detectable amounts of DNA. RNA concentration was determined by measuring UV absorbance at 260 nm with a SmartSpec Plus spectrophotometer (Bio-Rad, Hercules, CA, USA). The integrity and quality of the RNA were verified by agarose gel electrophoresis and calculation of the *A*_*260*_/*A*_*280*_ ratio.

### Gene cloning and sequencing

To isolate fragments of energy-related genes, degenerated oligonucleotides were constructed based on conserved sequence elements of *AtpB*, *AOX*, *UCP*, *AAC*, and *SnRK* from other species (Table 2). Using these oligonucleotides, amplification of fragments was carried out on a mixture of cDNA obtained from pericarp of litchi fruit stored for 0 and 2 days, because the genes displayed high expression at these stages in a preliminary analysis. The product of the first-strand cDNA was subjected to PCR amplification. The PCR product of the predicted size was purified, cloned into the pMD20-T vector (TaKaRa), and then transformed into *E. coli* DH5a cells (TaKaRa) in accordance with the manufacturer’s protocol. Plasmid DNA isolated from positive *E. coli* cells was digested with *Eco*RI and *Hin*dIII. The inserted DNA was sequenced by Sangon Biotech Co., Shanghai, China. The DNA fragments were extended by 3^′^- and 5^′^-RACE using the 3^′^-Full RACE Core Set version 2.0 and the 5^′^ RACE Kit (TaKaRa) with gene-specific primers. PCR products from 3^′^- or 5^′^-RACE were sequenced, and their sequence information was used to design gene-specific primers for full-length amplification. Full-length clones from each gene were sequenced in double strands. The resulting sequences were designated as *LcAtpB*, *LcAOX1*, *LcUCP1*, *LcAAC1*, and *LcSnRK2*, and were deposited in GenBank (http://www.ncbi.nlm.nih.gov) with the accession numbers JQ349005 (*LcAtpB*), JQ349006 (*LcAOX1*), JQ349009 (*LcUCP1*), JQ349007 (*LcAAC1*), and JQ349008 (*LcSnRK2*) (Table 3).

### Determination of gene transcript levels

Total RNA, extracted from the different plant tissues, was reverse-transcribed in 20 μL reaction mixture containing oligo d(T)_18_ primer using the Prime-Script™ RT-PCR Kit (TaKaRa, Japan) in accordance with the manufacturer’s protocol. Relative transcript abundance was analyzed by quantitative real-time PCR (qPCR) using the ABI 7500 Real-Time PCR System (Applied Biosystems, Carlsbad, CA, USA) and the LightCycler 480 SYBR Green I Master Mix (Roche Applied Science, http://www.roche-applied-science.com), in accordance with the manufacturer’s instructions under the following conditions: 30 s at 95°C, 40 cycles of 5 s at 95°C, and 34 s at 58°C. The primer pairs are listed in Table 4. The *LcACTIN* gene was used for quantitative normalization [65,66].

**Table 4:** Primers used for real-time PCR analysis

**Gene**	**Forward primer (5’ to 3’)**	**Reverse primer (5’ to 3’)**	**Product (bp)**
*LcAtpB*	GAGAGTTGGTTTGACTGCCCTAA	GAAGGCATTCTACCCAATAAGGC	141
*LcAOX1*	TCGGCTATCACTAAGATGTTGGTCA	ACAAGCACACACTCAACGGATTTAC	149
*LcUCP1*	AGTTGGCGGTGATTTTATTGGAG	GCATTCAAGGTCCCATAATAACG	177
*LcAAC1*	CTGAAGAATGAGGGTGCCAAGTC	CTAAAACAGCGAAAGGAATACCG	171
*LcSnRK2*	AGTTGAAGGACATCTGCTTGGAA	ATGCGAAAACCATACCTGTGTCA	153

See legend for Table 2 for names of genes.

### Bioinformatics and statistical analysis

Identification of nucleotide sequences from RT-PCR clones was carried out with the NCBI BLAST program (http://www.ncbi.nlm.nih.gov/BLAST). Alignments were made using Clustal X and Jalview software, and cladograms were constructed by the neighbor-joining method using the MEGA program and visualized with TreeView software. The theoretical isoelectric point (pI) and mass value for mature peptides were calculated using the PeptideMass program (http://us.expasy.org/tools/peptidemass.html).

The experiment was repeated twice during two consecutive growing seasons. Experiments for respiration intensity were repeated six times, whereas other experiments were repeated three times. The results are presented as the mean values ± standard deviation. Significant differences among different developmental stages, different storage times, and those between ATP-treated fruit and the untreated control were determined by one-way analysis of variance (ANOVA) using SPSS® version 13.0 (SPSS, Inc., Chicago, IL, USA). Statistical differences were assessed with a significance level of 5%. Graphs were drawn with SigmaPlot 9.0 and Excel 2003.

## Competing interests

The authors declare that they have no competing interests.

## Authors’ contributions

HW and ZJQ performed the experiments. SMM, YCZ and JWP largely contributed to the manuscript revision, HW and HXQ analyzed the data; YMJ and HXQ conceived and designed the experiment. HXQ wrote the paper. XWD participated in the design of the study. All authors have read and approved the final manuscript.

## Supplementary Material

Additional file 1 **Sequence alignment of LcAtpB and AtpBs from other plant species.** The alignment was made using CLUSTAL X software. Identical and similar amino acids are indicated by black and grey shading, respectively. The highly conserved ‘DELSEED’ motif is underlined. Abbreviations on the left of each sequence: At, *Arabidopsis thaliana*; St, *Solanum tuberosum*; Si, *Sesamum indicum*; Ma, *Melia azedarach*; and Zm, *Zea mays*. Click here for file

Additional file 2 **Cladogram of LcAtpB and AtpBs from other plant species.** The cladogram was constructed with the neighbor-joining method using MEGA software with default settings. Numbers at the nodes indicate bootstrap values. A scale of distance was shown at the bottom. The deduced amino acid sequences of plant AtpBs were obtained from the following sources, with their GenBank accession numbers enclosed in parentheses: *Koelreuteria paniculata*, KpAtpB (CAB89921.1); *Cneorum tricoccon*, CtAtpB (ACZ73599.1); *Citrus sinensis*, CsAtpB (YP_740482.1); *Melia azedarach*, MaAtpB (ABU75138.1); *Picrasma excelsa*, PeAtpB (ABU75177.1); *Sesamum indicum*, SiAtpB (CAB65433.1); *Solanum tuberosum*, StAtpB (ABB90048.1); *Arabidopsis thaliana,* AtAtpB (BAA84392.1); *Oryza sativa*, OsAtpB (NP_039390.1); and *Zea mays,* ZmAtpB (CAA60293.1). Click here for file

Additional file 3 **Sequence alignment of LcAOX1 and AOXs from other plant species.**The alignment was made using CLUSTAL X software. Identical and similar amino acids are indicated by black and grey shading, respectively. The key residues required for catalysis are numbered based on the *Litchi chinensis* AOX1 protein. Abbreviations on the left of each sequence: St, *Solanum tuberosum*; Ng, *Nicotiana glutinosa*; At, *Arabidopsis thaliana*; Pt, *Populus tremula* x *Populus tremuloides*; and Os, *Oryza sativa*. Click here for file

Additional file 4 **Cladogram of LcAOX1 and AOXs from other plant species.** The cladogram was constructed with the neighbor-joining method using the MEGA software with default settings. Numbers at the nodes indicate bootstrap values. A scale of distance was shown at the bottom. The deduced amino acid sequences of plant AOXs were obtained from the following sources, with their GenBank accession numbers enclosed in parentheses: *Nicotiana glutinosa*, NgAOX1a (ABU24346.1); *Solanum tuberosum*, StAOX (BAE92716.1); *Arabidopsis thaliana*, AtAOX1A (NP_188876.1), and AtAOX1B (NP_188875.1); *Citrus sinensis*, CsAOX (ACE95101.1); *Vigna unguiculata*, VuAOX1 (AAZ09196.1); *Gossypium hirsutum*, GhAOX (ABJ98721.1); *Populus tremula* x *Populus tremuloides*, PtAOX (CAB64356.1); *Daucus carota*, DcAOX1a (ABZ81227.2); *Nelumbo nucifera*, NnAOX1a (BAH56640.1); *Glycine max*, GmAOX1 (NP_001236166.1); *Oryza sativa*, OsAOX (BAA28774.1); *Triticum aestivum*, TaAOX (BAB88645.1); and *Zea mays*, ZmAOX (NP_001105180.1). Click here for file

Additional file 5 **Sequence alignment of LcUCP1 and UCPs from other plant species.** The alignment was made using CLUSTAL X software. Identical and similar amino acids are indicated by black and grey shading, respectively. Energy transfer protein signatures (ETPS) are underlined. Abbreviations on the left of each sequence: At, *Arabidopsis thaliana*; Rc, *Ricinus communis*; Zm, *Zea mays*; and Gm, *Glycine max*. Click here for file

Additional file 6 **Cladogram of LcUCP1 and UCPs from other plant species.** The cladogram was constructed with the neighbor-joining method using MEGA software with default settings. Numbers at the nodes indicate bootstrap values. A scale of distance was shown at the bottom. The deduced amino acid sequences of plant UCPs were obtained from the following sources, with their GenBank accession numbers enclosed in parentheses: *Arabidopsis thaliana*, AtUCP1 (NP_190979.1), AtUCP2 (NM_125287.4), and AtUCP5 (NP_179836.1); *Solanum tuberosum*, StUCP (CAA72107.1); *Glycine max*, GmUCP3 (XP_003516932.1); *Medicago truncatula*, MtUCP (AES86982.1); *Symplocarpus renifolius*, SrUCP (BAI49702.1); *Zea mays*, ZmUCP3 (NP_001182792.1); *Oryza sativa*, OsUCp (BAB40658.1); *Triticum aestivum*, TaUCP (BAB16385.1); and *Ricinus communis*, RcUCP (XM_002520396.1). Click here for file

Additional file 7 **Sequence alignment of LcAAC1 and AACs from other plant species.** The alignment was made using CLUSTAL X software. Identical and similar amino acids are indicated by black and grey shading, respectively. Asterisk indicates the mitochondria AAC signature motif (RRRMMM). Abbreviations on the left of each sequence: At, *Arabidopsis thaliana*; Rc, *Ricinus communis*; and Zm, *Zea mays*. Click here for file

Additional file 8 **Cladogram of LcAAC1 and AACs from other plant species.** The cladogram was constructed with the neighbor-joining method using MEGA software with default settings. Numbers at the nodes indicate bootstrap values. A scale of distance was shown at the bottom. The deduced amino acid sequences of plant AACs were obtained from the following sources, with their GenBank accession numbers enclosed in parentheses: *Ricinus communis*, RcAAC (XM_002531865.1); *Glycine max*, GmAAC1 (XP_003546882.1); *Zea mays*, ZmANT (CAA33742.1); *Arabidopsis thaliana*, AtAAC1 (NP_187470.1), and AtAAC2 (NM_121352.3); *Solanum lycopersicum*, SlANT (NP_001234018.1); *Medicago truncatula*, MtAAC (XP_003627715.1); and *Chlamydomonas incerta*, CiAAC (ABA01103.1). Click here for file

Additional file 9 **Sequence alignment of LcSnRK2 and SnRKs from other plant species.** The alignment was made using CLUSTAL X software. Identical and similar amino acids are indicated by black and grey shading, respectively. Regions 1–3 represent the ATP binding site, protein kinase activating signature, and C-terminal regulatory domain, respectively. Abbreviations on the left of each sequence: Rc, *Ricinus communis*; Gm, *Glycine max*; Zm, *Zea mays*; and At, *Arabidopsis thaliana*. Click here for file

Additional file 10 **Cladogram of LcSnRK2 and SnRKs from other plant species.** The cladogram was constructed with the neighbor-joining method using MEGA software with default settings. Numbers at the nodes indicate bootstrap values. A scale of distance was shown at the bottom. The deduced amino acid sequences of plant SnRKs were obtained from the following sources, with their GenBank accession numbers enclosed in parentheses: *Glycine max*, GmSAPK2 (XP_003519175.1); *Ricinus communis*, RcSAPK1 (XM_002513909.1), and RcSAPK3 (XP_002517501.1); *Zea mays*, ZmSnRK2.1 (ACG50005.1) and ZmSnRK2.2 (ACG50006.1); *Solanum lycopersicum*, SlSNF1 (NP_001234353.1); *Oryza sativ*, OsSAPK3 (BAD17999.1); *Arabidopsis thaliana*, AtSnRK1.3 (NM_123306.1), and AtSnRK2.2 (NM_001203118.1). Click here for file

Additional file 11 **Appearance of litchi fruit with or without exogenous ATP supply before storage at 25°C for 6 days.** (A) Untreated control; (B) treated with exogenous ATP. Numbers 0 to IV represent the pericarp browning scale: 0, no browning (excellent quality); I, slight browning; II, <1/4 browning; III, 1/4 to 1/2 browning, and IV, >1/2 browning (poor quality), respectively. Click here for file
