# Supplementary material for: Energy status of ripening and postharvest senescent fruit of litchi (Litchi chinensis Sonn.)
Source: BMC Plant Biol. 2013 Apr 2;13:55. doi: 10.1186/1471-2229-13-55 (PMC3636124; doi:10.1186/1471-2229-13-55)
Supplement: Additional file 1 — Sequence alignment of LcAtpB and AtpBs from other plant species. The alignment was made using CLUSTAL X software. Identical and similar amino acids are indicated by black and grey shading, respectively. The highly conserved ‘DELSEED’ motif is underlined. Abbreviations on the left of each sequence: At, Arabidopsis thaliana; St, Solanum tuberosum; Si, Sesamum indicum; Ma, Melia azedarach; and Zm, Zea mays. [file 1471-2229-13-55-S1.pdf]

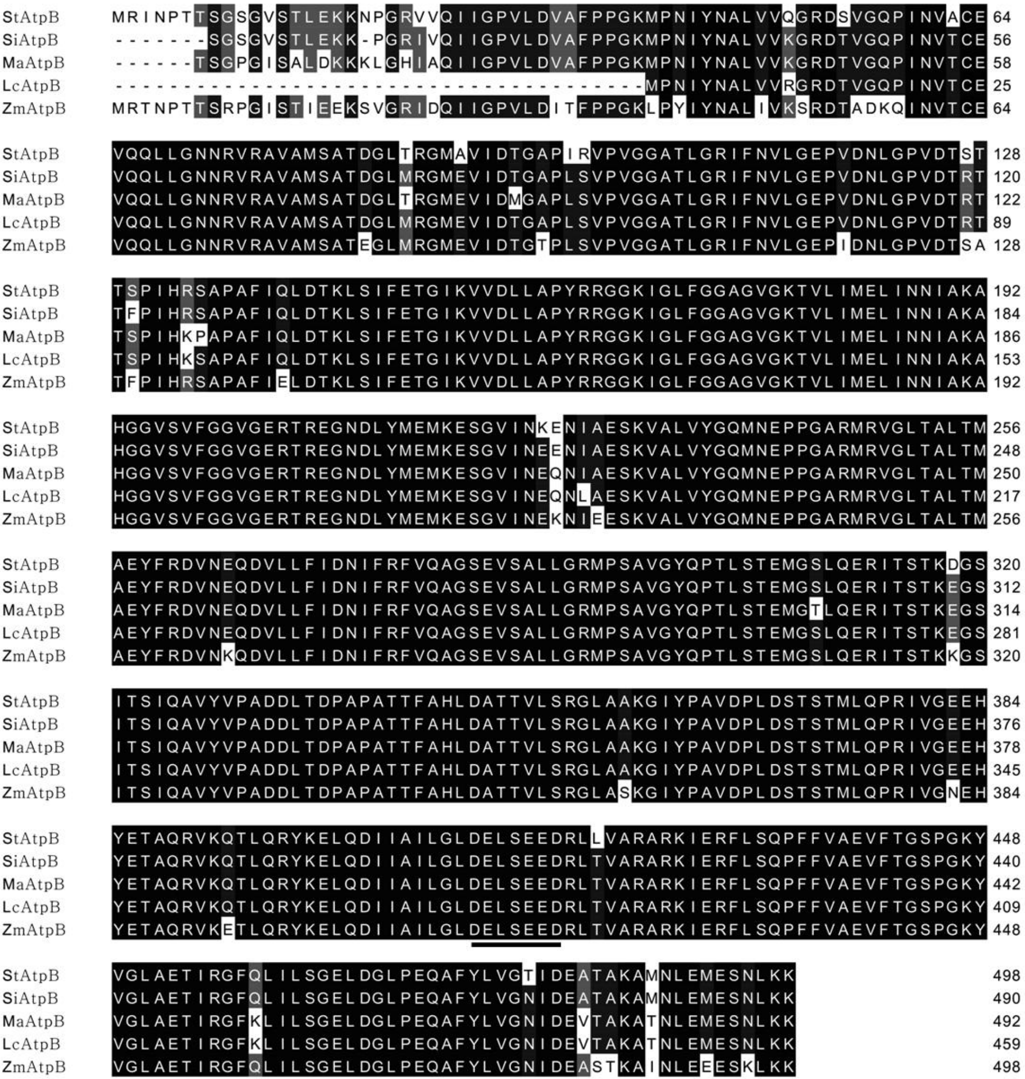
StAtpB MRINPTTSGSGVSTLEKKNPGRVVQIIGPVLDVAFPPGKMPNIYNALVVQGRDSVGQPINVACE 64
SiAtpB -------SGSGVSTLEKK-PGRIVQIIGPVLDVAFPPGKMPNIYNALVVKGRDTVGQPINVTCE 56
MaAtpB ------TSGPGISALDKKKLGHIAQIIGPVLDVAFPPGKMPNIYNALVVKGRDTVGQPINVTCE 58
LcAtpB ---------------------------------------MPNIYNALVVRGRDTVGQPINVTCE 25
ZmAtpB MRTNPTTSRPGISTIEEKSVGRIDQIIGPVLDITFPPGKLPYIYNALIVKSRDTADKQINVTCE 64
StAtpB VQQLLGNNRVRAVAMSATDGLTRGMAVIDTGAPIRVPVGGATLGRIFNVLGEPVDNLGPVDTST 128
SiAtpB VQQLLGNNRVRAVAMSATDGLMRGMEVIDTGAPLSVPVGGATLGRIFNVLGEPVDNLGPVDTRT 120
MaAtpB VQQLLGNNRVRAVAMSATDGLTRGMEVIDMGAPLSVPVGGATLGRIFNVLGEPVDNLGPVDTRT 122
LcAtpB VQQLLGNNRVRAVAMSATDGLMRGMEVIDTGAPLSVPVGGATLGRIFNVLGEPVDNLGPVDTRT 89
ZmAtpB VQQLLGNNRVRAVAMSATEGLMRGMEVIDTGTPLSVPVGGATLGRIFNVLGEPIDNLGPVDTSA 128
StAtpB TSPIHRSAPAFIQLDTKLSIFETGIKVVDLLAPYRRGGKIGLFGGAGVGKTVLIMELINNIAKA 192
SiAtpB TFPIHRSAPAFIQLDTKLSIFETGIKVVDLLAPYRRGGKIGLFGGAGVGKTVLIMELINNIAKA 184
MaAtpB TSPIHKPAPAFIQLDTKLSIFETGIKVVDLLAPYRRGGKIGLFGGAGVGKTVLIMELINNIAKA 186
LcAtpB TSPIHKSAPAFIQLDTKLSIFETGIKVVDLLAPYRRGGKIGLFGGAGVGKTVLIMELINNIAKA 153
ZmAtpB TFPIHRSAPAFIELDTKLSIFETGIKVVDLLAPYRRGGKIGLFGGAGVGKTVLIMELINNIAKA 192
StAtpB HGGVSVFGGVGERTREGNDLYMEMKESGVINKENIAESKVALVYGQMNEPPGARMRVGLTALTM 256
SiAtpB HGGVSVFGGVGERTREGNDLYMEMKESGVINEENIAESKVALVYGQMNEPPGARMRVGLTALTM 248
MaAtpB HGGVSVFGGVGERTREGNDLYMEMKESGVINEQNIAESKVALVYGQMNEPPGARMRVGLTALTM 250
LcAtpB HGGVSVFGGVGERTREGNDLYMEMKESGVINEQNLAESKVALVYGQMNEPPGARMRVGLTALTM 217
ZmAtpB HGGVSVFGGVGERTREGNDLYMEMKESGVINEKNIEESKVALVYGQMNEPPGARMRVGLTALTM 256
StAtpB AEYFRDVNEQDVLLFIDNIFRFVQAGSEVSALLGRMPSAVGYQPTLSTEMGSLQERITSTKDGS 320
SiAtpB AEYFRDVNEQDVLLFIDNIFRFVQAGSEVSALLGRMPSAVGYQPTLSTEMGSLQERITSTKEGS 312
MaAtpB AEYFRDVNEQDVLLFIDNIFRFVQAGSEVSALLGRMPSAVGYQPTLSTEMGTLQERITSTKEGS 314
LcAtpB AEYFRDVNEQDVLLFIDNIFRFVQAGSEVSALLGRMPSAVGYQPTLSTEMGSLQERITSTKEGS 281
ZmAtpB AEYFRDVNKQDVLLFIDNIFRFVQAGSEVSALLGRMPSAVGYQPTLSTEMGSLQERITSTKKGS 320
StAtpB ITSIQAVYVPADDLTDPAPATTFAHLDATTVLSRGLAAKGIYPAVDPLDSTSTMLQPRIVGEEH 384
SiAtpB ITSIQAVYVPADDLTDPAPATTFAHLDATTVLSRGLAAKGIYPAVDPLDSTSTMLQPRIVGEEH 376
MaAtpB ITSIQAVYVPADDLTDPAPATTFAHLDATTVLSRGLAAKGIYPAVDPLDSTSTMLQPRIVGEEH 378
LcAtpB ITSIQAVYVPADDLTDPAPATTFAHLDATTVLSRGLAAKGIYPAVDPLDSTSTMLQPRIVGEEH 345
ZmAtpB ITSIQAVYVPADDLTDPAPATTFAHLDATTVLSRGLASKGIYPAVDPLDSTSTMLQPRIVGNEH 384
StAtpB YETAQRVKQTLQRYKELQDIIAILGLDELSEEDRLLVARARKIERFLSQPFFVAEVFTGSPGKY 448
SiAtpB YETAQRVKQTLQRYKELQDIIAILGLDELSEEDRLTVARARKIERFLSQPFFVAEVFTGSPGKY 440
MaAtpB YETAQRVKQTLQRYKELQDIIAILGLDELSEEDRLTVARARKIERFLSQPFFVAEVFTGSPGKY 442
LcAtpB YETAQRVKQTLQRYKELQDIIAILGLDELSEEDRLTVARARKIERFLSQPFFVAEVFTGSPGKY 409
ZmAtpB YETAQRVKETLQRYKELQDIIAILGLDELSEEDRLTVARARKIERFLSQPFFVAEVFTGSPGKY 448
StAtpB VGLAETIRGFQLILSGELDGLPEQAFYLVGTIDEATAKAMNLEMESNLKK 498
SiAtpB VGLAETIRGFQLILSGELDGLPEQAFYLVGNIDEATAKAMNLEMESNLKK 490
MaAtpB VGLAETIRGFKLILSGELDGLPEQAFYLVGNIDEVTAKATNLEMESNLKK 492
LcAtpB VGLAETIRGFKLILSGELDGLPEQAFYLVGNIDEVTAKATNLEMESNLKK 459
ZmAtpB VGLAETIRGFQLILSGELDGLPEQAFYLVGNIDEASTKAINLEEESKLKK 498
