# Supplementary figures and images for: Energy status of ripening and postharvest senescent fruit of litchi (Litchi chinensis Sonn.)
Source: BMC Plant Biol. 2013 Apr 2;13:55. doi: 10.1186/1471-2229-13-55 (PMC3636124; doi:10.1186/1471-2229-13-55)

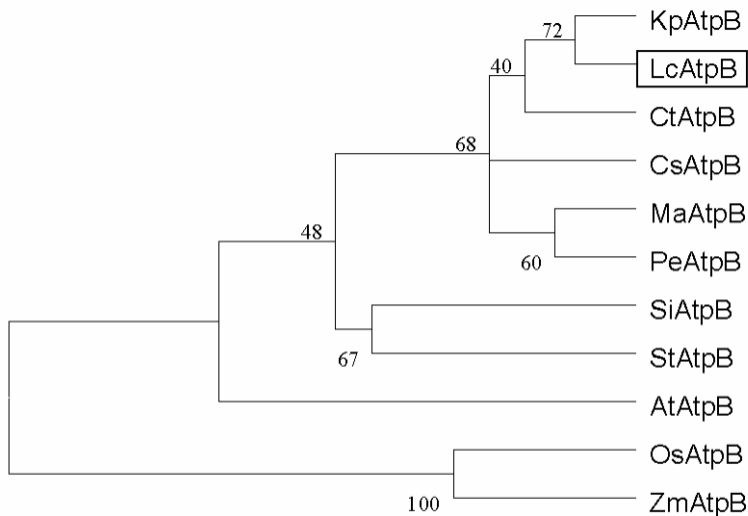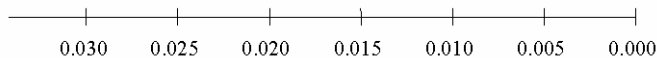

Supplement: Additional file 2 — Cladogram of LcAtpB and AtpBs from other plant species. The cladogram was constructed with the neighbor-joining method using MEGA software with default settings. Numbers at the nodes indicate bootstrap values. A scale of distance was shown at the bottom. The deduced amino acid sequences of plant AtpBs were obtained from the following sources, with their GenBank accession numbers enclosed in parentheses: Koelreuteria paniculata, KpAtpB (CAB89921.1); Cneorum tricoccon, CtAtpB (ACZ73599.1); Citrus sinensis, CsAtpB (YP_740482.1); Melia azedarach, MaAtpB (ABU75138.1); Picrasma excelsa, PeAtpB (ABU75177.1); Sesamum indicum, SiAtpB (CAB65433.1); Solanum tuberosum, StAtpB (ABB90048.1); Arabidopsis thaliana, AtAtpB (BAA84392.1); Oryza sativa, OsAtpB (NP_039390.1); and Zea mays, ZmAtpB (CAA60293.1). [file 1471-2229-13-55-S2.pdf]

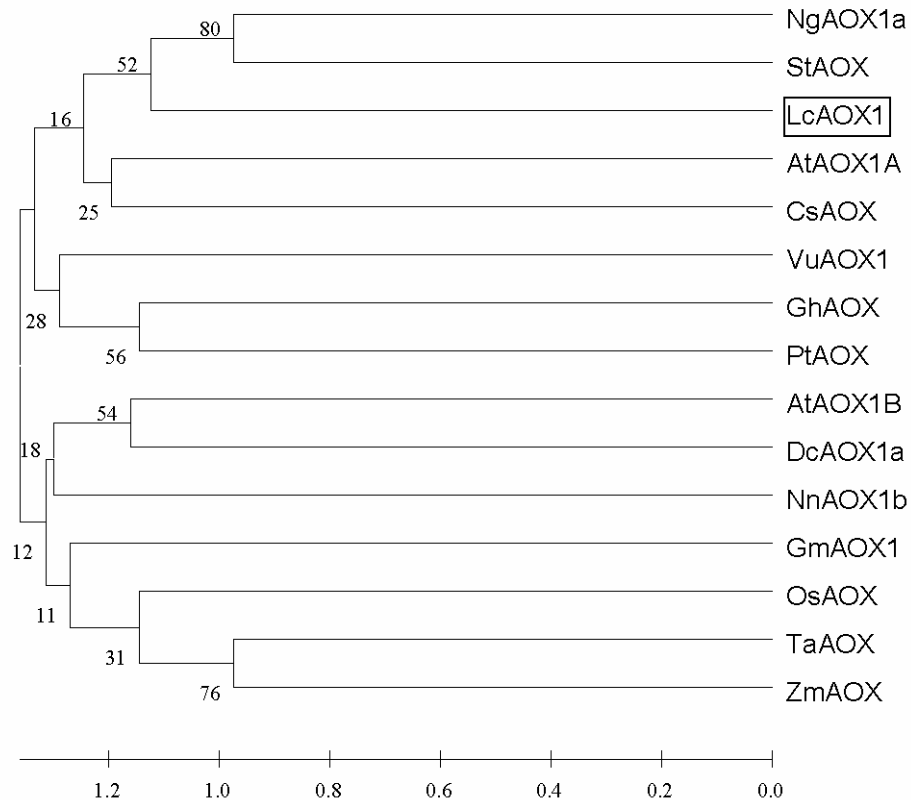

Supplement: Additional file 4 — Cladogram of LcAOX1 and AOXs from other plant species. The cladogram was constructed with the neighbor-joining method using the MEGA software with default settings. Numbers at the nodes indicate bootstrap values. A scale of distance was shown at the bottom. The deduced amino acid sequences of plant AOXs were obtained from the following sources, with their GenBank accession numbers enclosed in parentheses: Nicotiana glutinosa, NgAOX1a (ABU24346.1); Solanum tuberosum, StAOX (BAE92716.1); Arabidopsis thaliana, AtAOX1A (NP_188876.1), and AtAOX1B (NP_188875.1); Citrus sinensis, CsAOX (ACE95101.1); Vigna unguiculata, VuAOX1 (AAZ09196.1); Gossypium hirsutum, GhAOX (ABJ98721.1); Populus tremula x Populus tremuloides, PtAOX (CAB64356.1); Daucus carota, DcAOX1a (ABZ81227.2); Nelumbo nucifera, NnAOX1a (BAH56640.1); Glycine max, GmAOX1 (NP_001236166.1); Oryza sativa, OsAOX (BAA28774.1); Triticum aestivum, TaAOX (BAB88645.1); and Zea mays, ZmAOX (NP_001105180.1). [file 1471-2229-13-55-S4.pdf]

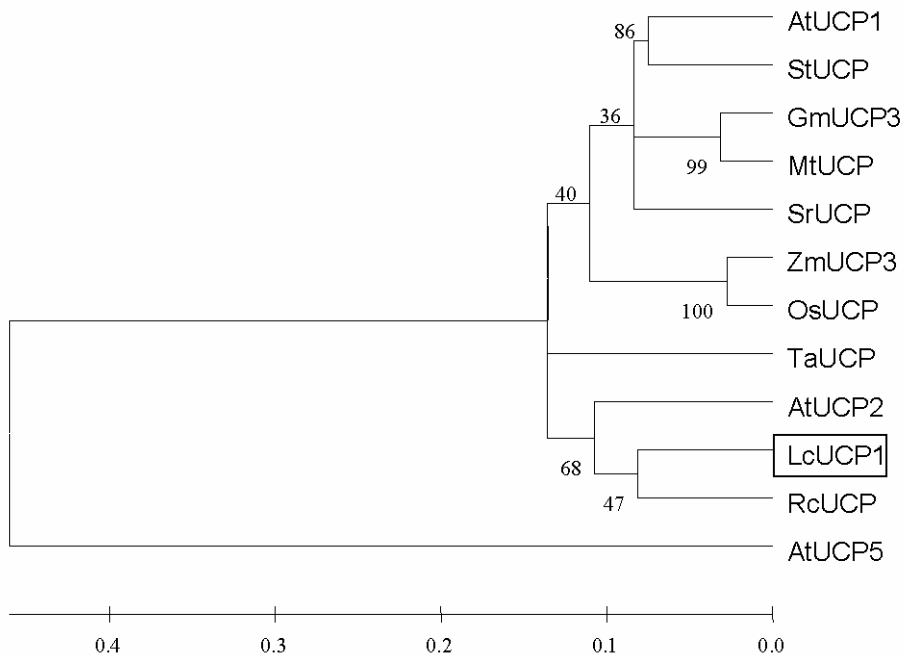

Supplement: Additional file 6 — Cladogram of LcUCP1 and UCPs from other plant species. The cladogram was constructed with the neighbor-joining method using MEGA software with default settings. Numbers at the nodes indicate bootstrap values. A scale of distance was shown at the bottom. The deduced amino acid sequences of plant UCPs were obtained from the following sources, with their GenBank accession numbers enclosed in parentheses: Arabidopsis thaliana, AtUCP1 (NP_190979.1), AtUCP2 (NM_125287.4), and AtUCP5 (NP_179836.1); Solanum tuberosum, StUCP (CAA72107.1); Glycine max, GmUCP3 (XP_003516932.1); Medicago truncatula, MtUCP (AES86982.1); Symplocarpus renifolius, SrUCP (BAI49702.1); Zea mays, ZmUCP3 (NP_001182792.1); Oryza sativa, OsUCp (BAB40658.1); Triticum aestivum, TaUCP (BAB16385.1); and Ricinus communis, RcUCP (XM_002520396.1). [file 1471-2229-13-55-S6.pdf]

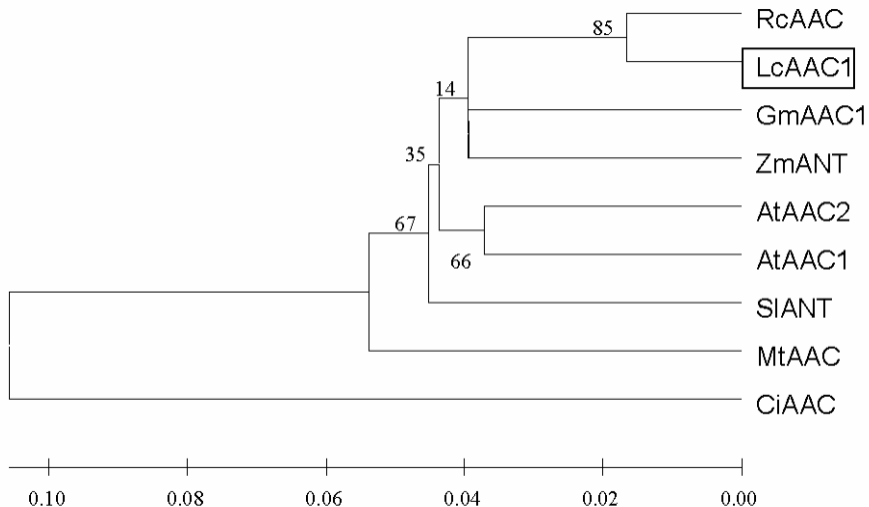

Supplement: Additional file 8 — Cladogram of LcAAC1 and AACs from other plant species. The cladogram was constructed with the neighbor-joining method using MEGA software with default settings. Numbers at the nodes indicate bootstrap values. A scale of distance was shown at the bottom. The deduced amino acid sequences of plant AACs were obtained from the following sources, with their GenBank accession numbers enclosed in parentheses: Ricinus communis, RcAAC (XM_002531865.1); Glycine max, GmAAC1 (XP_003546882.1); Zea mays, ZmANT (CAA33742.1); Arabidopsis thaliana, AtAAC1 (NP_187470.1), and AtAAC2 (NM_121352.3); Solanum lycopersicum, SlANT (NP_001234018.1); Medicago truncatula, MtAAC (XP_003627715.1); and Chlamydomonas incerta, CiAAC (ABA01103.1). [file 1471-2229-13-55-S8.pdf]

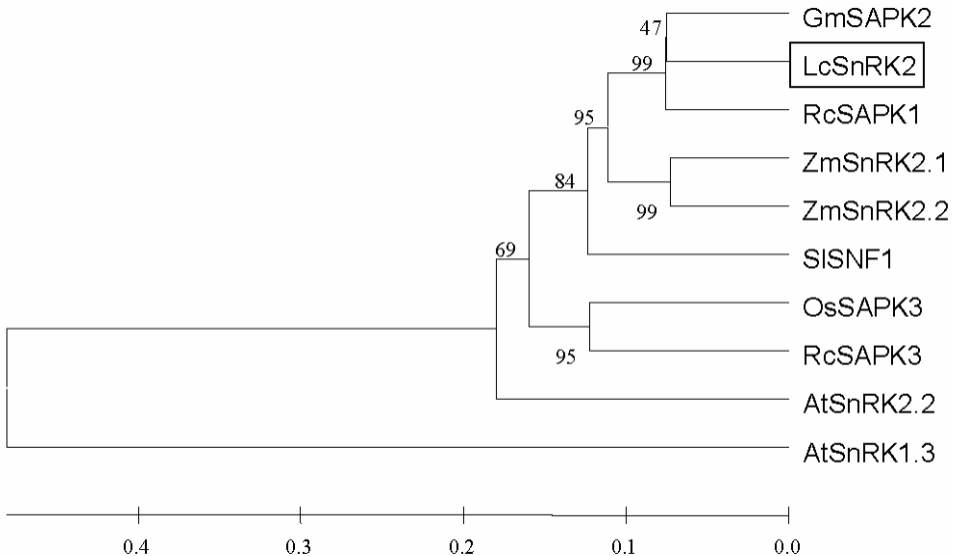

Supplement: Additional file 10 — Cladogram of LcSnRK2 and SnRKs from other plant species. The cladogram was constructed with the neighbor-joining method using MEGA software with default settings. Numbers at the nodes indicate bootstrap values. A scale of distance was shown at the bottom. The deduced amino acid sequences of plant SnRKs were obtained from the following sources, with their GenBank accession numbers enclosed in parentheses: Glycine max, GmSAPK2 (XP_003519175.1); Ricinus communis, RcSAPK1 (XM_002513909.1), and RcSAPK3 (XP_002517501.1); Zea mays, ZmSnRK2.1 (ACG50005.1) and ZmSnRK2.2 (ACG50006.1); Solanum lycopersicum, SlSNF1 (NP_001234353.1); Oryza sativ, OsSAPK3 (BAD17999.1); Arabidopsis thaliana, AtSnRK1.3 (NM_123306.1), and AtSnRK2.2 (NM_001203118.1). [file 1471-2229-13-55-S10.pdf]

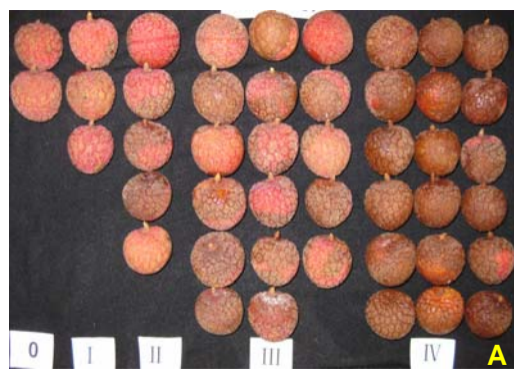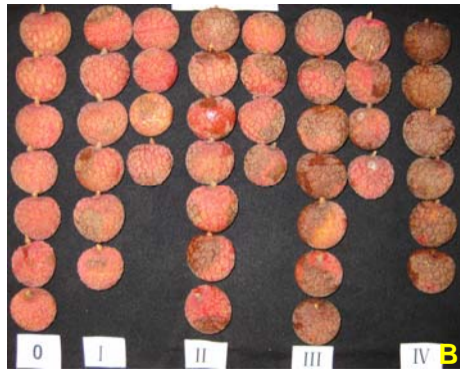

Supplement: Additional file 11 — Appearance of litchi fruit with or without exogenous ATP supply before storage at 25°C for 6 days. (A) Untreated control; (B) treated with exogenous ATP. Numbers 0 to IV represent the pericarp browning scale: 0, no browning (excellent quality); I, slight browning; II, <1/4 browning; III, 1/4 to 1/2 browning, and IV, >1/2 browning (poor quality), respectively. [file 1471-2229-13-55-S11.pdf]
