# Supplementary material for: Energy status of ripening and postharvest senescent fruit of litchi (Litchi chinensis Sonn.)
Source: BMC Plant Biol. 2013 Apr 2;13:55. doi: 10.1186/1471-2229-13-55 (PMC3636124; doi:10.1186/1471-2229-13-55)
Supplement: Additional file 3 — Sequence alignment of LcAOX1 and AOXs from other plant species.The alignment was made using CLUSTAL X software. Identical and similar amino acids are indicated by black and grey shading, respectively. The key residues required for catalysis are numbered based on the Litchi chinensis AOX1 protein. Abbreviations on the left of each sequence: St, Solanum tuberosum; Ng, Nicotiana glutinosa; At, Arabidopsis thaliana; Pt, Populus tremula x Populus tremuloides; and Os, Oryza sativa. [file 1471-2229-13-55-S3.pdf]

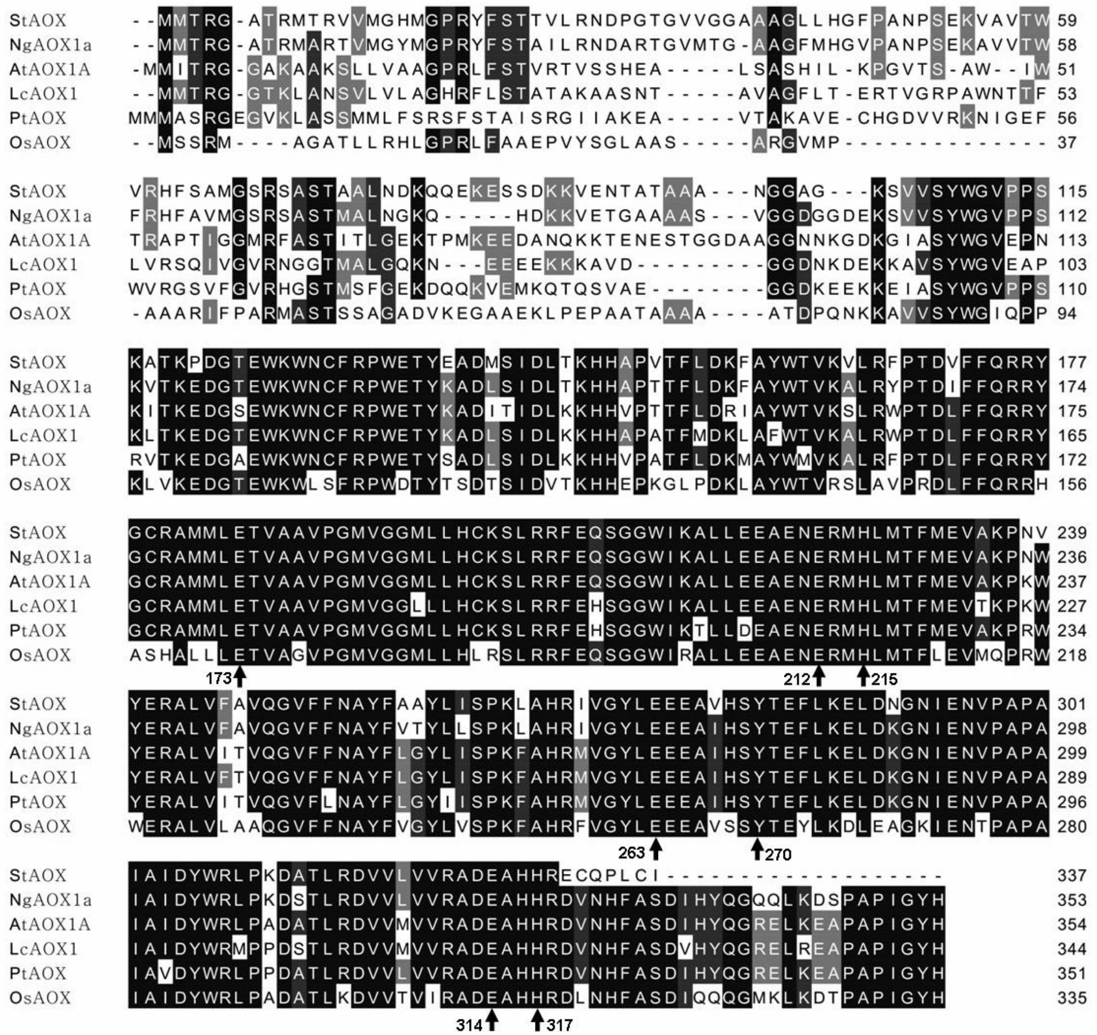
StAOX --MMTRG-ATRMTRVVMGHMGPRYFSTTVLRNDPGTGVVGGAAAGLLHGFPANPSEKVAVTW 59
NgAOX1a --MMTRG-ATRMARTVMGYMGPRYFSTAILRNDARTGVMTG-AAGFMHGVPANPSEKAVVTW 58
AtAOX1A -MMITRG-GAKAAKSLLVAAGPRLFSTVRTVSSHEA-----LSASHIL-KPGVTS-AW--IW 51
LcAOX1 --MMTRG-GTKLANSVLVLAGHRFLSTATAKAASNT-----AVAGFLT-ERTVGRPAWNTTF 53
PtAOX MMMASRGEGVKLASSMMLFSRSFSTAISRGIIAKEA-----VTAKAVE-CHGDVVRKNIGEF 56
OsAOX --MSSRM----AGATLLRHLGPRLFAAEPVYSGLAAS-----ARGVMP--------------- 37
StAOX VRHFSAMGSRSASTAALNDKQQEKESSDKKVENTATAAA---NGGAG---KSVVSYWGVPPS 115
NgAOX1a FRHFAVMGSRSASTMALNGKQ-----HDKKVETGAAAAS---VGGDGGDEKSVVSYWGVPPS 112
AtAOX1A TRAPTIGGMRFASTITLGEKTPMKEEDANQKKTENESTGGDAAGGNNKGDKGIASYWGVEPN 113
LcAOX1 LVRSQIVGVRNGGTMALGQKN---EEEEKKKAVD---------GGDNKDEKKAVSYWGVEAP 103
PtAOX WVRGSVFGVRHGSTMSFGEKDQQKVEMKQTQSVAE--------GGDKEEKKEIASYWGVPPS 110
OsAOX -AAARIFPARMASTSSAGADVKEGAAEKLPEPAATAAA----ATDPQNKKAVVSYWGIQPP 94
StAOX KATKPDGTEWKWNCFRPWETYEADMSIDLTKHHAPVTFLDKFAYWTVKVLRFPTDVFFQRRY 177
NgAOX1a KVTKEDGTEWKWNCFRPWETYKADLSIDLTKHHAPTTFLDKFAYWTVKALRYPTDIFFQRRY 174
AtAOX1A KITKEDGSEWKWNCFRPWETYKADITIDLKKHHVPTTFLDRIAYWTVKSLRWPTDLFFQRRY 175
LcAOX1 KLTKEDGTEWKWNCFRPWETYKADLSIDLKKHHAPATFMDKLAFWTVKALRWPTDLFFQRRY 165
PtAOX RVTKEDGAEWKWNCFRPWETYSADLSIDLKKHHVPATFLDKMAYWMVKALRFPTDLFFQRRY 172
OsAOX KLVKEDGTEWKWLSFRPWDTYTSDTSIDVTKHHEPKGLPDKLAYWTVRSLAVPRDLFFQRRH 156
StAOX GCRAMMLETVAAVPGMVGGMLLHCKSLRRFEQSGGWIKALLEEAENERMHLMTFMEVAKPNV 239
NgAOX1a GCRAMMLETVAAVPGMVGGMLLHCKSLRRFEQSGGWIKALLEEAENERMHLMTFMEVAKPNW 236
AtAOX1A GCRAMMLETVAAVPGMVGGMLLHCKSLRRFEQSGGWIKALLEEAENERMHLMTFMEVAKPKW 237
LcAOX1 GCRAMMLETVAAVPGMVGGLLLHCKSLRRFEHSGGWIKALLEEAENERMHLMTFMEVTKPKW 227
PtAOX GCRAMMLETVAAVPGMVGGMLLHCKSLRRFEHSGGWIKTLLDEAENERMHLMTFMEVAKPRW 234
OsAOX ASHALLLETVAGVPGMVGGMLLHLRSLRRFEQSGGWIRALLEEAENERMHLMTFLEVMQPRW 218
173
212
215
StAOX YERALVFAVQGVFFNAYFAAYLISPKLAHRIVGYLEEEAVHSYTEFLKELDNGNIENVPAPA 301
NgAOX1a YERALVFAVQGVFFNAYFVTYLLSPKLAHRIVGYLEEEAIHSYTEFLKELDKGNIENVPAPA 298
AtAOX1A YERALVITVQGVFFNAYFLGYLISPKFAHRMVGYLEEEAIHSYTEFLKELDKGNIENVPAPA 299
LcAOX1 YERALVFTVQGVFFNAYFLGYLISPKFAHRMVGYLEEEAIHSYTEFLKELDKGNIENVPAPA 289
PtAOX YERALVITVQGVFLNAYFLGYIISPKFAHRMVGYLEEEAIHSYTEFLKELDKGNIENVPAPA 296
OsAOX WERALVLAAQGVFFNAYFVGYLVSPKFAHRFVGYLEEEAVSSYTEYLKDLEAGKIENTPAPA 280
263
270
StAOX IAIDYWRLPKDATLRDVVLVVRADEAHHRECQPLCI------------------- 337
NgAOX1a IAIDYWRLPKDSTLRDVVLVVRADEAHHRDVNHFASDIHYQGQQLKDSPAPIGYH 353
AtAOX1A IAIDYWRLPADATLRDVVMVVRADEAHHRDVNHFASDIHYQGRELKEAPAPIGYH 354
LcAOX1 IAIDYWRMPPDSTLRDVVMVVRADEAHHRDVNHFASDVHYQGRELREAPAPIGYH 344
PtAOX IAVDYWRLPPDATLRDVVLVVRADEAHHRDVNHFASDIHYQGRELKEAPAPIGYH 351
OsAOX IAIDYWRLPADATLKDVVTVIRADEAHHRDLNHFASDIQQQGMKLKDTPAPIGYH 335
314
317
