# Supplementary material for: Energy status of ripening and postharvest senescent fruit of litchi (Litchi chinensis Sonn.)
Source: BMC Plant Biol. 2013 Apr 2;13:55. doi: 10.1186/1471-2229-13-55 (PMC3636124; doi:10.1186/1471-2229-13-55)
Supplement: Additional file 5 — Sequence alignment of LcUCP1 and UCPs from other plant species. The alignment was made using CLUSTAL X software. Identical and similar amino acids are indicated by black and grey shading, respectively. Energy transfer protein signatures (ETPS) are underlined. Abbreviations on the left of each sequence: At, Arabidopsis thaliana; Rc, Ricinus communis; Zm, Zea mays; and Gm, Glycine max. [file 1471-2229-13-55-S5.pdf]

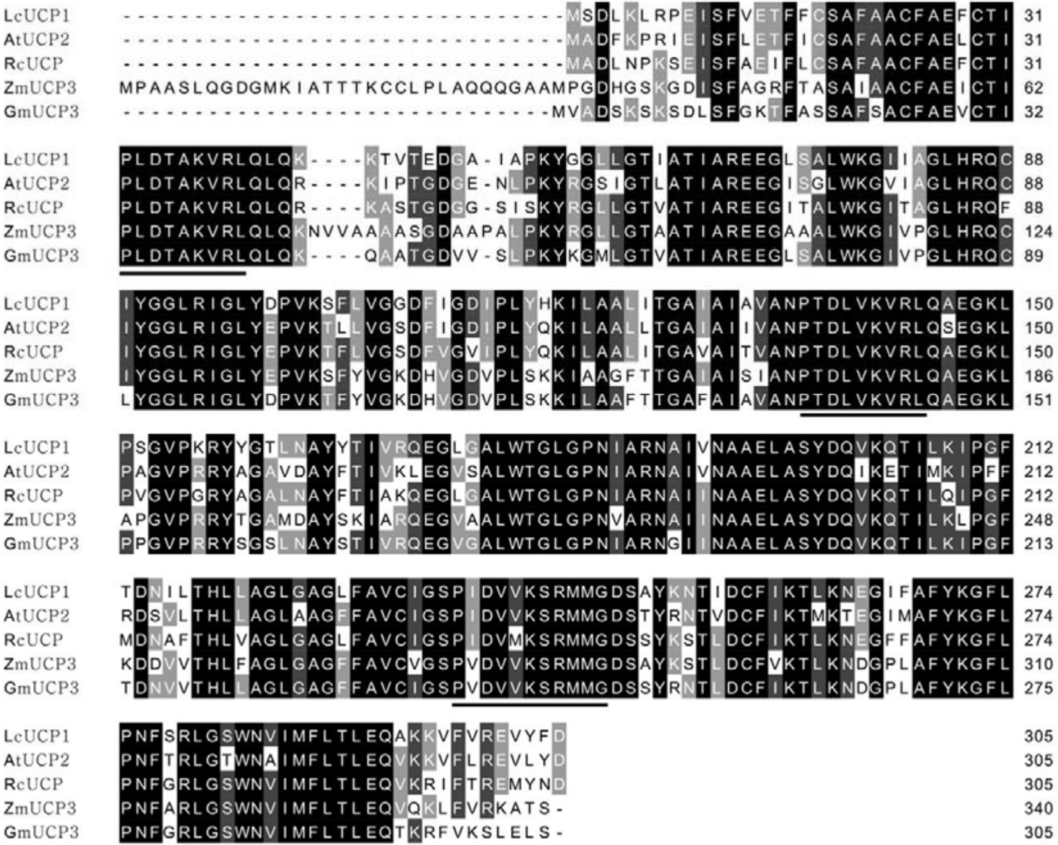

LcUCP1 -------------------------------MSDLKLRPEISFVETFFCSAFAACFAEFCTI 31
AtUCP2 -------------------------------MADFKPRIEISFLETFICSAFAACFAELCTI 31
RcUCP -------------------------------MADLNPKSEISFAEIFLCSAFAACFAEFCTI 31
ZmUCP3 MPAASLQGDGMKIATTTKCCLPLAQQQGAAMPGDHGSKGDISFAGRFTASAIAACFAEICTI 62
GmUCP3 ------------------------------MVADSKSKSDLSFGKTFASSAFSACFAEVCTI 32
LcUCP1 PLDTAKVRLQLQK----KTVTEDGA-IAPKYGGLLGTIATIAREEGLSALWKGIIAGLHRQC 88
AtUCP2 PLDTAKVRLQLQR----KIPTGDGE-NLPKYRGSIGTLATIAREEGISGLWKGVIAGLHRQC 88
RcUCP PLDTAKVRLQLQR----KASTGDGG-SISKYRGLLGTVATIAREEGITALWKGITAGLHRQF 88
ZmUCP3 PLDTAKVRLQLQKNVVAAAASGDAAPALPKYRGLLGTAATIAREEGAAALWKGIVPGLHRQC 124
GmUCP3 PLDTAKVRLQLQK----QAATGDVV-SLPKYKGMLGTVATIAREEGLSALWKGIVPGLHRQC 89
LcUCP1 IYGGLRIGLYDPVKSFLVGGDFIGDIPLYHKILAALITGAIAIAVANPTDLVKVRLQAEGKL 150
AtUCP2 IYGGLRIGLYEPVKTLLVGSDFIGDIPLYQKILAALLTGAIAIIVANPTDLVKVRLQSEGKL 150
RcUCP IYGGLRIGLYEPVKTFLVGSDFVGVIPLYQKILAALITGAVAITVANPTDLVKVRLQAEGKL 150
ZmUCP3 IYGGLRIGLYEPVKSFYVGKDHVGDVPLSKKIAAGFTTGAIAISIANPTDLVKVRLQAEGKL 186
GmUCP3 LYGGLRIGLYDPVKTFYVGKDHVGDVPLSKKILAAFTTGAFAIAVANPTDLVKVRLQAEGKL 151
LcUCP1 PSGVPKRYYGTLNAYYTIVRQEGLGALWTGLGPNIARNAIVNAAELASYDQVKQTILKIPGF 212
AtUCP2 PAGVPRRYAGAVDAYFTIVKLEGVSALWTGLGPNIARNAIVNAAELASYDQIKETIMKIPFF 212
RcUCP PVGVPGRYAGALNAYFTIAKQEGLGALWTGLGPNIARNAIINAAELASYDQVKQTILQIPGF 212
ZmUCP3 APGVPRRYTGAMDAYSKIARQEGVAALWTGLGPNVARNAIINAAELASYDQVKQTILKLPGF 248
GmUCP3 PPGVPRRYSGSLNAYSTIVRQEGVGALWTGLGPNIARNGIINAAELASYDQVKQTILKIPGF 213
LcUCP1 TDNILTHLLAGLGAGLFAVCIGSPIDVVKSRMMGDSAYKNTIDCFIKTLKNEGIFAFYKGFL 274
AtUCP2 RDSVLTHLLAGLAAGFFAVCIGSPIDVVKSRMMGDSTYRNTVDCFIKTMKTEGIMAFYKGFL 274
RcUCP MDNAFTHLVAGLGAGLFAVCIGSPIDVMKSRMMGDSSYKSTLDCFIKTLKNEGFFAFYKGFL 274
ZmUCP3 KDDVVTHLFAGLGAGFFAVCVGSPVDVVKSRMMGDSAYKSTLDCFVKTLKNDGPLAFYKGFL 310
GmUCP3 TDNVVTHLLAGLGAGFFAVCIGSPVDVVKSRMMGDSSYRNTLDCFIKTLKNDGPLAFYKGFL 275
LcUCP1 PNFSRLGSWNVIMFLTLEQAKKVFVREVYFD 305
AtUCP2 PNFTRLGTWNAIMFLTLEQVKKVFLREVLYD 305
RcUCP PNFGRLGSWNVIMFLTLEQVKRIFTREMYND 305
ZmUCP3 PNFARLGSWNVIMFLTLEQVQKLFVRKATS- 340
GmUCP3 PNFGRLGSWNVIMFLTLEQTKRFVKSLELS- 305
