# Supplementary material for: Energy status of ripening and postharvest senescent fruit of litchi (Litchi chinensis Sonn.)
Source: BMC Plant Biol. 2013 Apr 2;13:55. doi: 10.1186/1471-2229-13-55 (PMC3636124; doi:10.1186/1471-2229-13-55)
Supplement: Additional file 7 — Sequence alignment of LcAAC1 and AACs from other plant species. The alignment was made using CLUSTAL X software. Identical and similar amino acids are indicated by black and grey shading, respectively. Asterisk indicates the mitochondria AAC signature motif (RRRMMM). Abbreviations on the left of each sequence: At, Arabidopsis thaliana; Rc, Ricinus communis; and Zm, Zea mays. [file 1471-2229-13-55-S7.pdf]

```
AtAAC2  ---------------------------------MVEQTQHPTILQKVSGQLLSSSVSQDIRGY  30
AtAAC1  ---------------------------------MVDQVQHPTIAQKAAGQFMRSSVSKDVQ--  28
RcAAC   ---------------------------------MADQVQYPSVMQKVAGQYLHSSLSQDFQGF  30
LcAAC1  MEKKQSERRRQTDQPVTSCHQSLFSLSFSGKVAMDQVQHPSVMQKVASQLLHSSLTHDVRSC  62
ZmANT   ---------------------------------MADQANQPTVLHKLGGQFHLRSIISEGVRA  30

AtAAC2  ASASKRPATYQKHAAYGNYSNAAFQYPLVAAS------QIATTTSPVFVQAPGEKGFTNFAI  86
AtAAC1  -VGYQRPSMYQRHATYGNYSNAAFQFPPTSR-------MLATTASPVFVQTPGEKGFTNFAL  82
RcAAC   DGSLQRPALYQRR-AYGNYTNAAFQYPMGCA----DLSVVPTTASSVCVQAPSEKGFAGFAI  87
LcAAC1  DGAFNRPALYQRR-TYGNYSNAAFHPVVRACGATSDMSMVASTASPVFVQAPSEKGFAGFAI  123
ZmANT   RNICPSVSSYERRFATRNYMTQSLWGPSMSVSG---GINVPVMQTPLCANAPAEKGGKNFMI  89

AtAAC2  DFMMGGVSAAVSKTAAAPIERVKLLIQNQDEMLKAGRLTEPYKGIRDCFGRTIRDEGIGSLW  148
AtAAC1  DFLMGGVSAAVSKTAAAPIERVKLLIQNQDEMIKAGRLSEPYKGIGDCFGRTIKDEGFGSLW  144
RcAAC   DFLMGGVSAAVSKTAAAPIERVKLLIQNQDEMIKSGRLSEPYKGIGDCFKRTIKDEGFGSLW  149
LcAAC1  DFLMGGVSAAVSKTAAAPIERVKLLIQNQDEMIKTGRLSEPYKGIGDCFKRTMKDEGIVSLW  185
ZmANT   DFMMGGVSAAVSETAAAPIERVKLLIQNQDEMIKSGRLSEPYKGIVDCFKRTIKDEGFSSLW  151

AtAAC2  RGNTANVIRYFPTQALNFAFKDYFKRLFNFKKDKDGYWKWFAGNLASGGAAGASSLLFVYSL  210
AtAAC1  RGNTANVIRYFPTQALNFAFKDYFKRLFNFKKDRDGYWKWFAGNLASGGAAGASSLLFVYSL  206
RcAAC   RGNTANVIRYFPTQALNFAFKDYFKRLFNFKKDKDGYWKWFAGNLASGGAAGASSLLFVYSL  211
LcAAC1  RGNTANVIRYFPTQALNFAFKDYFKRLFNFKKDKDGYWKWFAGNLASGGAAGASSLLFVYSL  247
ZmANT   RGNTANVIRYFPTQALNFAFKDYFKRLFNFKKDRDGYWKWFAGNLASGGAAGASSLFFVYSL  213

AtAAC2  DYARTRLANDSKSAKKGGGERQFNGLVDVYKKTLKSDGIAGLYRGFNISCAGIIVYRGLYFG  272
AtAAC1  DYARTRLANDAKAAKKGGGGRQFDGLVDVYRKTLKTDGIAGLYRGFNISCVGIIVYRGLYFG  268
RcAAC   DYARTRLANDAKAAKKGG-ERQFNGLVDVYRKTLASDGIAGLYRGFNISCVGIIVYRGLYFG  272
LcAAC1  DYARTRLANDAKAAKKGG-ERQFNGLVDVYRKTMKSDGIAGLYRGFNISCVGIIVYRGLYFG  308
ZmANT   DYARTRLANDAKAAKGGG-ERQFNGLVDVYRKTLKSDGIAGLYRGFNISCVGIIVYRGLYFG  274

AtAAC2  LYDSVKPVLLTGDLQDSFFASFALGWLITNGAGLASYPIDTVRRRMMMTSGEAVKYKSSFDA  334
AtAAC1  LYDSVKPVLLTGDLQDSFFASFALGWVITNGAGLASYPIDTVRRRMMMTSGEAVKYKSSLDA  330
RcAAC   MYDSLKPVVLTGNLQDSFFASFALGWLITNGAGLASYPIDTVRRRMMMTSGEAVKYRGSLDA  334
LcAAC1  MYDSLKPVVLTGSLQDSFFASFALGWLITNGAGLASYPIDTVRRRMMMTSGEAVKYKSSLDA  370
ZmANT   LYDSIKPVVLTGNLQDNFFASFALGWLITNGAGLASYPIDTVRRRMMMTSGEAVKYKSSLDA  336

AtAAC2  FSQIVKKEGAKSLFKGAGANILRAVAGAGVLAGYDKLQLIVFGKKYGSGGA-  385
AtAAC1  FKQILKNEGAKSLFKGAGANILRAVAGAGVLSGYDKLQLIVFGKKYGSGGA-  381
RcAAC   FSQILKNEGAKSLFKGAGANILRAVAGAGVLAGYDKLQLIVFGKKYGSGGA-  385
LcAAC1  FSQILKNEGAKSLFKGAGANILRAVAGAGVLAGYDKLQLIVFGKKYGSGGGA  422
ZmANT   FQQILKKEGPKSLFKGAGANILRAIAGAGVLSGYDQLQILFFGKKYGSGGA-  387
```
