# Supplementary material for: Energy status of ripening and postharvest senescent fruit of litchi (Litchi chinensis Sonn.)
Source: BMC Plant Biol. 2013 Apr 2;13:55. doi: 10.1186/1471-2229-13-55 (PMC3636124; doi:10.1186/1471-2229-13-55)
Supplement: Additional file 9 — Sequence alignment of LcSnRK2 and SnRKs from other plant species. The alignment was made using CLUSTAL X software. Identical and similar amino acids are indicated by black and grey shading, respectively. Regions 1–3 represent the ATP binding site, protein kinase activating signature, and C-terminal regulatory domain, respectively. Abbreviations on the left of each sequence: Rc, Ricinus communis; Gm, Glycine max; Zm, Zea mays; and At, Arabidopsis thaliana. [file 1471-2229-13-55-S9.pdf]

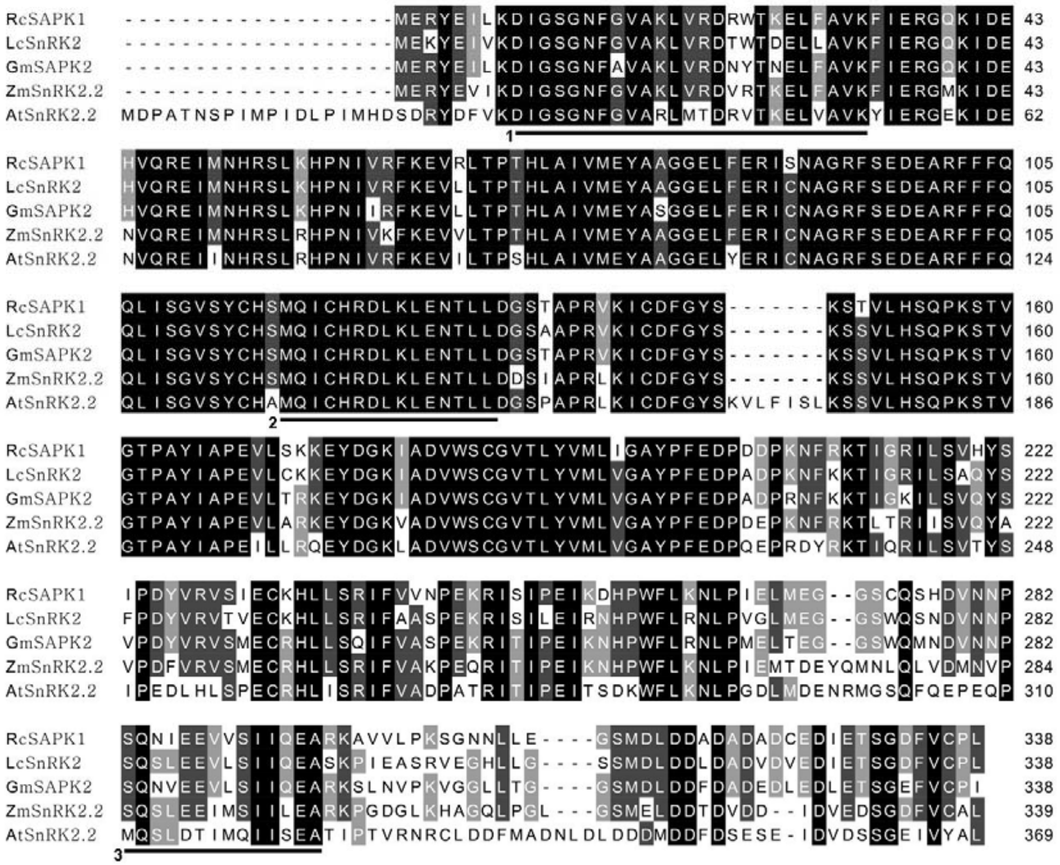
RcSAPK1 -------------------MERYEILKDIGSGNFGVAKLVRDRWTKELFAVKFIERGQKIDE 43
LcSnRK2 -------------------MEKYEIVKDIGSGNFGVAKLVRDTWTDELLAVKFIERGQKIDE 43
GmSAPK2 -------------------MERYEILKDIGSGNFAVAKLVRDNYTNELFAVKFIERGQKIDE 43
ZmSnRK2.2 -------------------MERYEVIKDIGSGNFGVAKLVRDVRTKELFAVKFIERGMKIDE 43
AtSnRK2.2 MDPATNSPIMPIDLPIMHDSDRYDFVKDIGSGNFGVARLMTDRVTKELVAVKYIERGEKIDE 62
1
RcSAPK1 HVQREIMNHRSLKHPNIVRFKEVRLTPTHLAIVMEYAAGGELFERISNAGRFSEDEARFFFQ 105
LcSnRK2 HVQREIMNHRSLKHPNIVRFKEVLLTPTHLAIVMEYAAGGELFERICNAGRFSEDEARFFFQ 105
GmSAPK2 HVQREIMNHRSLKHPNIIRFKEVLLTPTHLAIVMEYASGGELFERICNAGRFSEDEARFFFQ 105
ZmSnRK2.2 NVQREIMNHRSLRHPNIVKFKEVVLTPTHLAIVMEYAAGGELFERICNAGRFSEDEARFFFQ 105
AtSnRK2.2 NVQREIINHRSLRHPNIVRFKEVILTPSHLAIVMEYAAGGELYERICNAGRFSEDEARFFFQ 124
RcSAPK1 QLISGVSYCHSMQICHRDLKLENTLLDGSTAPRVKICDFGYS-------KSTVLHSQPKSTV 160
LcSnRK2 QLISGVSYCHSMQICHRDLKLENTLLDGSAAPRVKICDFGYS-------KSSVLHSQPKSTV 160
GmSAPK2 QLISGVSYCHSMQICHRDLKLENTLLDGSTAPRVKICDFGYS-------KSSVLHSQPKSTV 160
ZmSnRK2.2 QLISGVSYCHSMQICHRDLKLENTLLDDSIAPRLKICDFGYS-------KSSVLHSQPKSTV 160
AtSnRK2.2 QLISGVSYCHAMQICHRDLKLENTLLDGSPAPRLKICDFGYSKVLFISLKSSVLHSQPKSTV 186
2
RcSAPK1 GTPAYIAPEVLSKKEYDGKIADVWSCGVTLYVMLIGAYPFEDPDDPKNFRKTIGRILSVHYS 222
LcSnRK2 GTPAYIAPEVLCKKEYDGKIADVWSCGVTLYVMLVGAYPFEDPADPKNFKKTIGRILSAQYS 222
GmSAPK2 GTPAYIAPEVLTRKEYDGKIADVWSCGVTLYVMLVGAYPFEDPADPRNFKKTIGKILSVQYS 222
ZmSnRK2.2 GTPAYIAPEVLARKEYDGKVADVWSCGVTLYVMLVGAYPFEDPDEPKNFRKTLTRIISVQYA 222
AtSnRK2.2 GTPAYIAPEILLRQEYDGKLADVWSCGVTLYVMLVGAYPFEDPQEPRDYRKTIQRILSVTYS 248
RcSAPK1 IPDYVRVSIECKHLLSRIFVVNPEKRISIPEIKDHPWFLKNLPIELMEG--GSCQSHDVNNP 282
LcSnRK2 FPDYVRVTVECKHLLSRIFAASPEKRISILEIRNHPWFLRNLPVGLMEG--GSWQSNDVNNP 282
GmSAPK2 VPDYVRVSMECRHLLSQIFVASPEKRITIPEIKNHPWFLRNLPMELTEG--GSWQMNDVNNP 282
ZmSnRK2.2 VPDFVRVSMECRHLLSRIFVAKPEQRITIPEIKNHPWFLKNLPIEMTDEYQMNLQLVDMNVP 284
AtSnRK2.2 IPEDLHLSPECRHLISRIFVADPATRITIPEITSDKWFLKNLPGDLMDENRMGSQFQEPEQP 310
RcSAPK1 SQNIEEVVSIIQEARKAVVLPKSGNNLLE----GSMDLDDADADADCEDIETSGDFVCPL 338
LcSnRK2 SQSLEEVLSIIQEASKPIEASRVEGHLLG----SSMDLDDLDADVDVEDIETSGDFVCPL 338
GmSAPK2 SQNVEEVLSIIQEARKSLNVPKVGGLLTG----GSMDLDDFDADEDLEDLETSGEFVCPI 338
ZmSnRK2.2 SQSLEEIMSIILEARKPGDGLKHAGQLPGL---GSMELDDTDVDD--IDVEDSGDFVCAL 339
AtSnRK2.2 MQSLDTIMQIISEATIPTVRNRCLDDFMADNLDLDDDMDDFDSESE-IDVDSSGEIVYAL 369
3
